# Visceral Adipose Tissue of Prediabetic and Diabetic Females Shares a Set of Similarly Upregulated microRNAs Functionally Annotated to Inflammation, Oxidative Stress and Insulin Signaling

**DOI:** 10.3390/antiox10010101

**Published:** 2021-01-12

**Authors:** Justyna Strycharz, Adam Wróblewski, Andrzej Zieleniak, Ewa Świderska, Tomasz Matyjas, Monika Rucińska, Lech Pomorski, Piotr Czarny, Janusz Szemraj, Józef Drzewoski, Agnieszka Śliwińska

**Affiliations:** 1 Department of Medical Biochemistry, Medical University of Lodz, 92-215 Lodz, Poland; adam.wroblewski@stud.umed.lodz.pl (A.W.); ewa.swiderska@umed.lodz.pl (E.Ś.); piotr.czarny@umed.lodz.pl (P.C.); janusz.szemraj@umed.lodz.pl (J.S.); 2 Department of Structural Biology, Medical University of Lodz, 90-752 Lodz, Poland; andrzej.zieleniak@umed.lodz.pl; 3 Clinical Department of General and Oncological Surgery, Medical University of Lodz, 92-213 Lodz, Poland; tobistm@gmail.com (T.M.); monika.rucinska@umed.lodz.pl (M.R.); lech.pomorski@umed.lodz.pl (L.P.); 4 Central Teaching Hospital of the Medical University of Lodz, 92-213 Lodz, Poland; jozef.drzewoski@umed.lodz.pl; 5 Department of Nucleic Acid Biochemistry, Medical University of Lodz, 92-213 Lodz, Poland

**Keywords:** microRNAs, visceral adipose tissue, visceral adipocytes, hyperglycemia, diabetes, impaired fasting glucose, sex differences, oxidative stress, inflammation, insulin signaling

## Abstract

Hypertrophic and hypoxic visceral adipose tissue (VAT) secretes proinflammatory cytokines promoting insulin resistance (IR), prediabetes and type 2 diabetes (T2DM) microRNAs (miRNAs) are markers of metabolic disorders regulating genes critical for e.g., inflammation, glucose metabolism, and antioxidant defense, with raising diagnostic value. The aim of the current study was to evaluate whether hyperglycemia is able to affect the expression of selected miRNAs in VAT of prediabetic (IFG) and diabetic (T2DM) patients vs. normoglycemic (NG) subjects using qPCR. Statistical analyses suggested that miRNAs expression could be sex-dependent. Thus, we determined 15 miRNAs as differentially expressed (DE) among NG, T2DM, IFG females (miR-10a-5p, let-7d-5p, miR-532-5p, miR-127-3p, miR-125b-5p, let-7a-5p, let-7e-5p, miR-199a-3p, miR-365a-3p, miR-99a-5p, miR-100-5p, miR-342-3p, miR-146b-5p, miR-204-5p, miR-409-3p). Majority of significantly changed miRNAs was similarly upregulated in VAT of female T2DM and IFG patients in comparison to NG subjects, positively correlated with FPG and HbA1c, yet, uncorrelated with WHR/BMI. Enrichment analyses indicated involvement of 11 top DE miRNAs in oxidative stress, inflammation and insulin signaling. Those miRNAs expression changes could be possibly associated with low-grade chronic inflammation and oxidative stress in VAT of hyperglycemic subjects.

## 1. Introduction

Type 2 diabetes (T2DM) constitutes a burden to modern society and manifests as a slowly process of gradual loss of glucose taken up by cells in an insulin-dependent way [1]. Long before T2DM is easily diagnosed, plasma glucose levels are above the reference range being called prediabetic state [2]. While there are 2 types of prediabetes (impaired fasting glucose (IFG), impaired glucose tolerance (IGT)), each of them is a risk factor for T2DM. Prediabetes frequently coexists with metabolic syndrome (MetS), thus showing imbalance not only of glucose, but also lipid metabolism.

Prediabetes and T2DM are inseparably connected with excessive visceral adiposity [3]. Hypertrophic and hypoxic visceral adipose tissue (VAT) is a huge, easily lymphocytes- and macrophages-infiltrated endocrine organ that produces and secretes predominantly proinflammatory set of cytokines into the bloodstream [4]. Indeed, the progression from normoglycemic to prediabetic, and diabetic state is associated with inflammation, which can be monitored via expression changes in inflammatory markers, including some novel ones like EN-RAGE and IL-17 [5]. VAT-specific expression of proinflammatory molecules is increased in comparison to subcutaneous adipose tissue (SAT) in T2DM patients, and related to insulin action and fasting glucose [6]. This further confirms a direct connection among visceral adiposity, insulin resistance (IR) and risk for T2DM [6]. Inflammation is also interrelated with oxidative stress, while the latter is pronouncedly marked in subjects with elevated and fluctuating blood glucose levels [7]. Hyperglycemia (HG) evokes elevation of reactive oxygen species (ROS) due to several well-known mechanisms i.e., (formation of advanced glycation end (AGE) products, activation of DAG/PKC signaling pathway and elevation of flux into hexosamine, polyol and the glyceraldehyde autoxidation pathways) connected with increased rate of mitochondrial metabolism, in this way perpetuating the vicious cycle of IR [8]. Hypertrophied VAT is also a source of ROS due to hypoxia, imbalance between activity and expression levels of antioxidant and prooxidant enzymes, impairment of numerous signaling pathways, macrophages infiltration and M1 polarization, all being associated with low-grade chronic inflammation.

Oxidative stress and inflammation are critical regulators of expression of sophisticated epigenetic modifiers—miRNAs in metabolic disorders [9]. miRNAs are short non-coding RNAs capable of regulating expression of numerous target genes. Hyperglycemia-dysregulated miRNAs affect insulin sensitive tissues by regulating molecules of insulin signaling pathway (e.g., Irs1, Irs2, Akt, Pten, Glut4, mTOR), inflammation (e.g., Tlr4, NfκB, IKK-β, IL−6), lipid metabolism (e.g., Sirt1), prooxidant (e.g., Nox) and antioxidant genes (e.g., Sod1, Nrf2, FoxO) and more [9]. Studies also suggest that miRNAs influence expression of adipokines and their receptors in obesity-related disorder [10]. Moreover, miRNAs in white adipose tissue (WAT) are becoming more and more regarded as biomarkers and therapeutic targets in tissues affected also by obesity [11]. This could be associated with the fact that miRNAs produced by adipose depots are easily transported into the bloodstream, then being able to affect metabolism of distant tissues [11]. For instance, miR-155-5p secreted with exosomes via AT macrophages in obese mice and transferred to lean mice evoked IR and glucose intolerance [12]. Many studies are now being focused on evaluation of serum miRNAs as possible markers enabling earlier prediction of transition from healthy to prediabetic and diabetic stage, however, still little is known about miRNAs in VAT [13,14,15]. In order to evaluate the impact of hyperglycemia on the expression of those important epigenetic molecules, we investigated relative expression of 75 selected miRNAs in VAT of normoglycemic, prediabetic and diabetic subjects.

## 2. Materials and Methods

### 2.1. Study Subjects and Ethics

Thirty-eight unrelated male and female Pole subjects aged 50–81 were admitted to Clinical Department of General and Oncological Surgery at The Central Teaching Hospital of the Medical University of Lodz for planned laparoscopic procedure (e.g., cholecystectomy) between October 2017 and January 2020. The study protocol was approved by the Ethics Committee of the Medical University of Lodz, Poland (no. RNN/287/13/KE). All at least 50-year-old study subjects were informed about the protocol for the study and provided the written consent for the collection of VAT sample from visceral peritoneum and sample of peripheral venous blood collected after at least 8 h of overnight fasting. Acute inflammation, neoplasms and medical history of neoplasms were exclusion criteria. The probands were classified as diabetic (N = 13), based on their prior diagnosis and therapy (metformin and/or flozins and/or sulfonylurea derivatives and/or insulin). Normoglycemic (N = 13) and prediabetic (N = 12, impaired fasting glucose type (IFG)) subjects were diagnosed in accordance with 2020 American Diabetes Association (ADA) criteria: FPG (fasting plasma glucose) ranging from 100 to 125 mg/dL and HbA1c (glycated hemoglobin) in the range of 5.7–6.4% for prediabetes (IFG) [16]. In this study, we compared miRNAs expression profile of subjects from three groups and denoted them as follows: NG (normoglycemic), IFG (those with impaired fasting glucose) and T2DM (diabetic) subjects.

### 2.2. Biochemical and Anthropometric Measurements

Data collection included: body mass, height, waist and hip circumference, which were used to calculate body mass index (BMI) and waist-hip ratio (WHR). Blood sample analysis involved measurement of FPG, fasting insulin (FI) concentration, HbA1c, total cholesterol (TC), high-density lipoprotein cholesterol (HDL-C), and triglycerides (TG). Low-density lipoprotein cholesterol (LDL-C) was calculated via Friedewald formula. Moreover, above parameters allowed for calculation of IR-associated parameters such as homeostasis model assessment for insulin resistance (HOMA-IR) and TG to HDL-C ratio (TG/HDL) [17].

### 2.3. VAT Sample Processing and Total RNA Isolation

Approximately 1 g of VAT was collected into sterile tubes containing PBS (Sigma Aldrich, Saint Louis, MO, USA) and immediately weighed and split into smaller samples. 250 mg of VAT samples were immediately processed with 700 μL of Lysis/Binding Buffer and frozen at −80 °C for further processing. Total RNA was isolated using mirVana™ miRNA Isolation Kit (Invitrogen™, Carlsbad, CA, USA) according to the manufacturer’s instructions and supplied by Life Technologies (Vilnius, Lithuania). Concentration and purity of freshly isolated total RNA were measured by Pico100 Spectrophotometer (Picodrop). RNA samples reaching 1.8–2.1 (A260/A280) were chosen for gene expression profiling.

### 2.4. Molecules Selection

The complete list of 78 miRNAs and 4 small nuclear RNAs (snRNAs) is provided in Table S1. Selection of the set of molecules was based on: (i) preliminary data obtained via previously used TLDA cards on 1 healthy and 1 diabetic subject, (ii) literature search for miRNAs associated with T2DM, IR, obesity in all insulin-responsive tissues, (iii) providing a broad panel of candidate reference genes (literature search), (iv) results of our previous research on hyperglycemia-exposed visceral pre/adipocytes [18,19].

### 2.5. Reverse Transcription and Expression Profiling

The methodology was described in detail previously [18]. Briefly, reverse transcription (15 μL) was carried out in Tpersonal Thermocycler (Biometra, Göttingen, Germany) in the following conditions: preheating for 30 min at 16 °C, cDNA synthesis for 30 min at 42 °C, RT inactivation for 5 min at 85 °C, and cooling at 4 °C, using TaqMan™ Total MicroRNA Reverse Transcription Kit (Applied Biosystems), Custom RT Pool (Applied Biosystems) and 1000 ng of total RNA, as indicated in the protocol for Custom TaqMan^®^ Array MicroRNA Cards (Applied Biosystems). Expression analysis was performed using Custom TaqMan^®^ Array MicroRNA Cards including internal negative control (hsa-miR-4267-242640_mat). Briefly, cDNA obtained in the RT reaction, TaqMan^®^ Universal PCR Master Mix II, no UNG (Applied Biosystems) and RNAse-free water were used for PCR mixture, which was loaded into two ports of a customized MicroRNA TLDA Card for each study subject. Subsequently, each card was centrifuged and sealed to perform quantitative PCR in 7900HT Fast Real-Time PCR System (Applied Biosystems; Thermo Fisher Scientific, Inc., Waltham, MA, USA)) in conditions indicated by the manufacturer. miR-596 was undetected in great majority of samples, and therefore, excluded from further analysis. The relative expression of 75 miRNAs was determined using the ΔCt method (Ct_miRNA_ − Ct_reference genes_), based on duplicate results. The pair of the best endogenous control genes (miR-93-5p and miR-17-5p) was chosen experimentally with Bestkeeper, excluding snRNAs due to controversy indicated in literature [20].

### 2.6. Functional Enrichment Analysis

Biological significance of DE miRNAs was assessed in silico with miEAA 2.0 [21] and miRSystem [22]. In miEAA 2.0, we performed an overrepresentation analysis with FDR correction and 0.05 significance level using “Annotations derived over miRTarBase (Gene Ontology)” and “Pathways (miRWalk)” while results were presented in a form of wordcloud of categories (top 100 by *p* value). Functional annotation in miRSystem was conducted on KEGG, BIOCARTA, PATHWAY INTERACTION DATABASE (human only) AND REACTOME (human only) with records sorted by empirical *p* value.

### 2.7. Statistical Analysis

Shapiro-Wilk’s and Levene’s tests were used for testing of normality of data distribution and equality of variance, respectively, affecting the way of data expression. Kruskal-Wallis one-way analysis of variance (ANOVA) by ranks was used to evaluate differences in age, biochemical and anthropometric parameters among three groups of study participants due to lack of normality of data distribution for all tested parameters. Significance of differences in miRNAs expression among three groups was tested with one-way ANOVA (Tukey’s HSD post hoc test) and the multi-class SAM (Significance Analysis of Microarrays) with False Discovery Rate (FDR) adjustment. Power calculations for each miRNA were provided in Table S2. Two-way ANOVA was performed to evaluate whether miRNAs expression changes could be also associated with sex. Univariate correlation among expression levels of individual miRNAs and each of biochemical and anthropometric parameters was performed with Spearman’s rank correlation and the correlation heatmap was generated with MeV software [23]. Euclidean hierarchical clustering (Ward’s algorithm), multi-class SAM, Sparse Partial Least Squares-Discriminant Analysis (sPLS-DA) with leave-one-out cross-validation (LOOCV), Receiver Operating Characteristic Curve (ROC) with area under the curve (AUC) biomarker analyses (optimal cutoff calculated with closest to top-left corner method) were conducted with Metaboanalyst 4.0 on the autoscaled data [24]. Autoscaling was depicted in Figures S1–S4. Other analyses were performed with STATISTICA 10.0 software (Statsoft, Tulsa, OK, USA) and GraphPad Prism. *p* ≤ 0.05 was considered as significant.

## 3. Results

### 3.1. Description of Study Participants 

**Figure 1:**
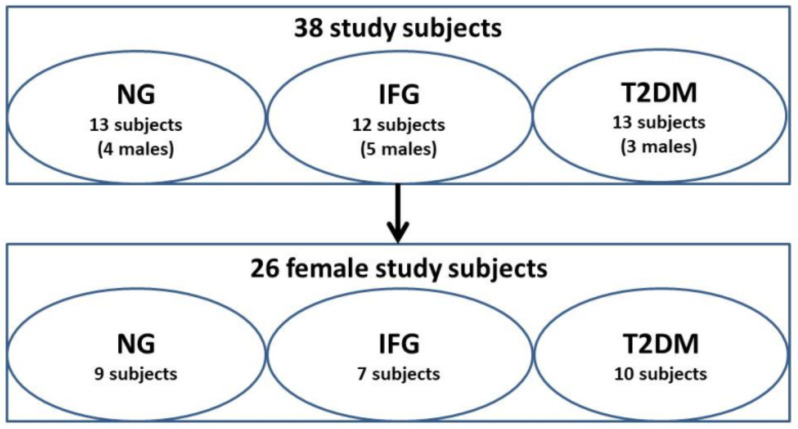
Study groups.

Thirty-eight age- and BMI-matched subjects (12 males and 26 females) were enrolled into the current study (Table 1, Figure 1). Weight, height, waist and hip circumference, WHR, FI, HOMA-IR and HDL levels did not differ among groups, however, analyses for FI and HOMA-IR were performed only for 5 diabetic subjects (Table 1). HbA1c was elevated among T2DM and IFG patients and in comparison to NG ones. FPG levels were also significantly elevated in IFG and T2DM patients when compared to healthy ones. We also observed decreased levels of TC and LDL in IFG patients in relation to NG ones and equaled levels of those parameters in T2DM and NG subjects. This could be associated with lipid-lowering treatment (unrecorded in our study). Moreover, T2DM patients had substantially higher levels of TG as compared to IFG ones. 

Considering female study participants, we observed that T2DM patients were characterized by higher values of FPG, HOMA-IR, HbA1c, TG and TG/HDL in relation to NG subjects (Table 1). However, HOMA-IR was significantly lower in IFG group than in females with T2DM. Regarding male subjects, those suffering from T2DM had substantially increased levels of FPG and HbA1c. There were no statistical differences between the rest of the analyzed parameters among NG, IFG and T2DM groups in male and female subgroups of study participants. Comparisons of FI and HOMA-IR were not performed due to insufficient number of diabetic male study subjects.

### 3.2. The Expression of the Set of miRNAs Is Changed in VAT of T2DM and IFG Patients in Comparison to NG Subjects, Yet Slightly or Uncorrelated with Parameters of Glucose Metabolism (FPG, HbA1c, FI, HOMA-IR and TG/HDL)

**Figure 2:**
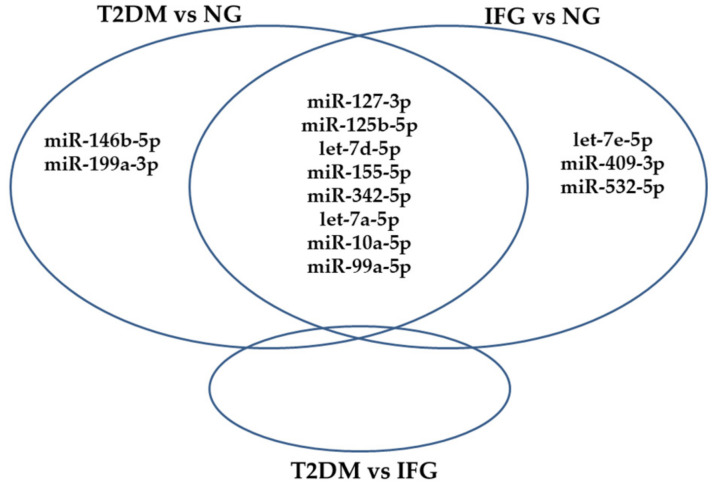
Venn diagram of significantly changed (upregulated) miRNAs (FDR < 0.2) between T2DM, IFG and NG subjects (N = 38) in VAT, based on one-way ANOVA and Tukey’s HSD post hoc test. (*p* ≤ 0.05).

**Figure A1:**
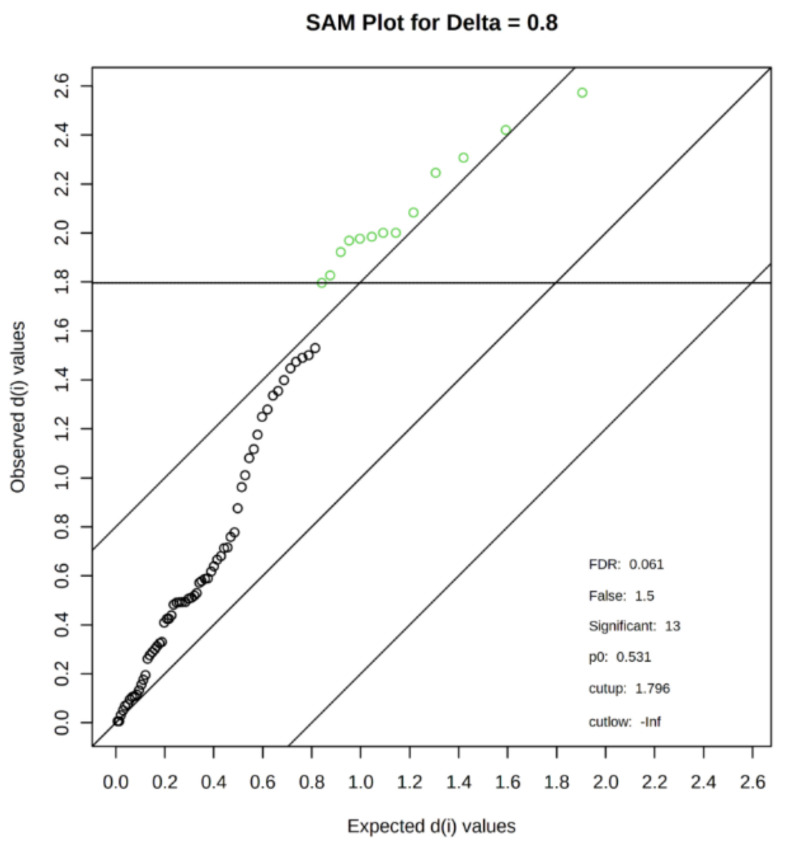
Results of SAM analysis for all study subjects (N = 38).

**Figure A2:**
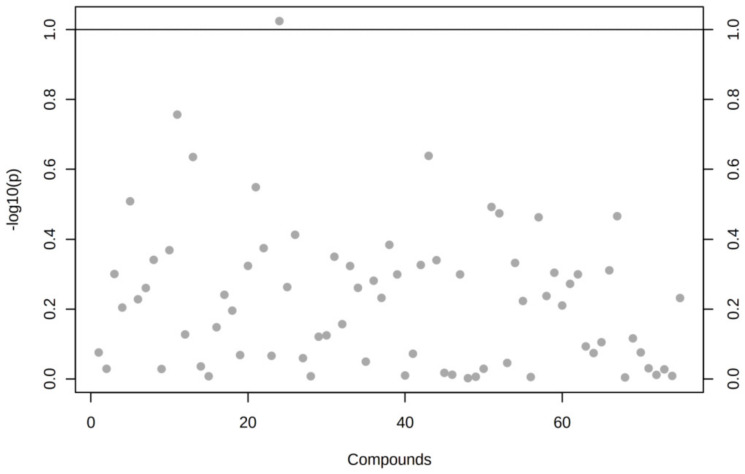
Graphical presentation of t-test analysis (FDR < 0.1) performed between T2DM and IFG study subjects (N = 38) by Metaboanalyst 4.0. No significantly changed miRNAs were reported.

We found that 13 miRNAs were DE among T2DM, IFG and NG subjects in VAT according to one-way ANOVA (FDR < 0.2) and SAM (FDR < 0.1) (Table 2, Figure A1). Importantly, expression changes of none of tested miRNAs met the criteria of FDR < 0.05. All DE miRNAs with regard to intergroup comparisons were detailed in Venn diagram in Figure 2. Interestingly, Tukey’s HSD post hoc test applied on one-way ANOVA results indicated significant upregulation of 8 miRNAs (miR-127-3p, miR-125b-5p, let-7d-5p, miR-155-5p, miR-342-5p, let-7a-5p, miR-10a-5p, miR-99a-5p) in VAT of IFG and T2DM patients as compared to NG ones (Figure 2). Moreover, expression of 2 sets of miRNAs was typically changed only in VAT of either T2DM (miR-146b-5p, miR-199a-3p) or IFG (let-7e-5p, miR-409-3p, miR-532-5p) patients in comparison to NG ones (Figure 2). Interestingly, none of tested miRNAs was significantly changed among T2DM and IFG patients in VAT (Figure 2 and Figure A2).

While we observed some moderate positive correlations among expression of miRNAs and mainly glucose metabolism-associated biochemical parameters, none of them passed FDR correction (0.1) (Table S3).

### 3.3. The Expression of the miRNAs in VAT Appears to Be Connected with Sex-Specific Differences

#### 3.3.1. Two-Way ANOVA

**Figure A3:**
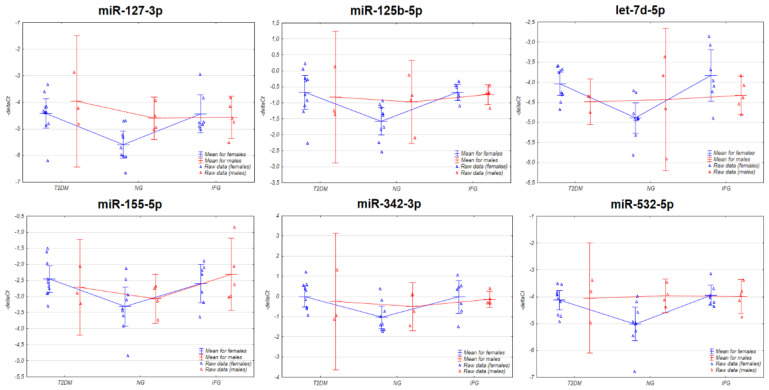
Plots for miR-127-3p, miR-125b-5p, let-7d-5p, miR-155-5p, miR-342-3p and miR-532-3p showing mean -deltaCt values with regard to studied group and sex for all study subjects (N = 38).

**Figure A4:**
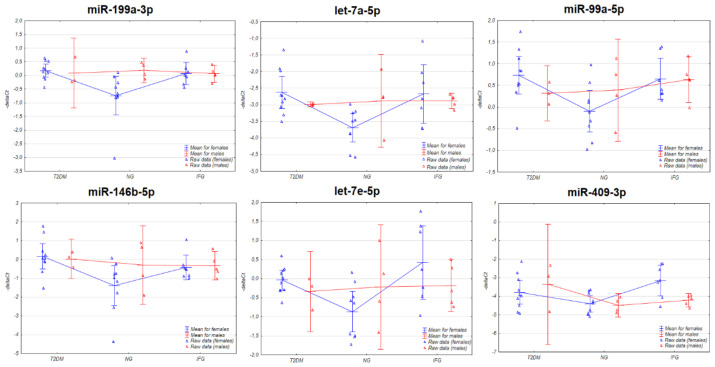
Plots for miR-127-3p, miR-125b-5p, let-7d-5p, miR-155-5p, miR-342-3p and miR-532-3p showing mean-deltaCt values with regard to studied group and sex for all study subjects (N = 38).

**Figure A5:**
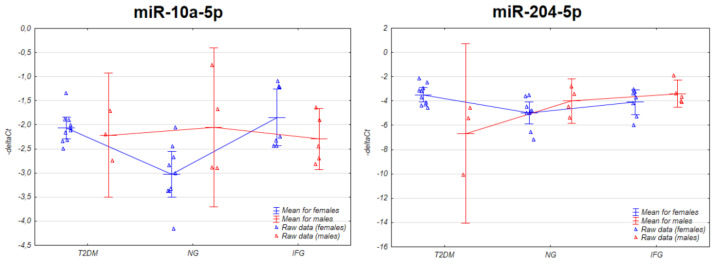
Plots for miR-10a-5p and miR-204-5p showing mean -deltaCt values with regard to studied group and sex for all study subjects (N = 38). Two-way ANOVA indicated significant interaction between study group and sex for both molecules (*p* < 0.02).

Lack of miRNAs expression changes and correlations meeting the criteria of FDR < 0.1 made us speculate about the possible confounders. Therefore, we evaluated expression plots for all DE miRNAs with regard to sex (Figure A3, Figure A4 and Figure A5). Despite the fact that the number of male subjects was limited, we noticed that only some miRNAs did not express tendency for sex-specific differences. For instance, miR-155-5p showed identical pattern of expression in male and female subjects (Figure A3). Interestingly, two-way ANOVA indicated significant interaction between sex and study group for expression changes of miR-10a-5p (*p* < 0.02) (Figure A5). Additionally, we presented the plot for miR-204-5p as a prominent example visualizing the impact of sex on miRNAs expression in VAT of T2DM patients according to two-way ANOVA (interaction between study group and sex with *p* = 0.0011) (Figure A5).

#### 3.3.2. Euclidean Hierarchical Clustering

**Figure 3:**
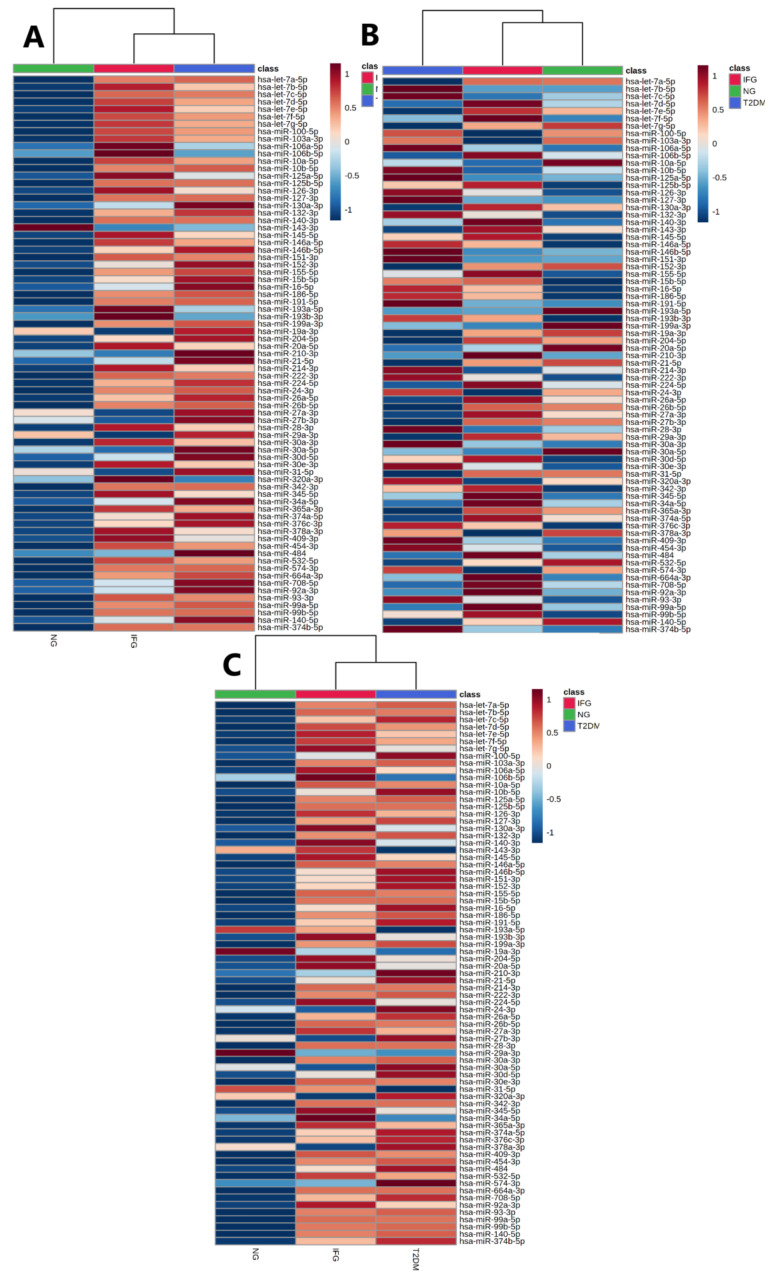
Heatmaps of Euclidean hierarchical clustering with Ward’s distance measure algorithm and group averages performed for all tested miRNAs for female (**A**), N = 26, male (**B**), N = 12 and all study subjects (**C**), N = 38.

We visualized expression profile of all tested miRNAs separately in female, male and all study participants via Euclidean hierarchical clustering (Figure 3A–C). As expected, miRNAs levels were substantially different between females and males, being further suggested by clustering. Namely, analyses indicated similarity of miRNAs expression profiles among IFG and T2DM females and their separation from NG females (Figure 3A). In contrast, male patients with IFG were grouped with NG ones, while those with T2DM formed a separate cluster (Figure 3B). Due to substantially higher number of female (N = 26) over male (N = 12) subjects, the heatmap for all study participants was highly similar to one generated for females. The results of above analyses should be taken with great caution due to occurrence of possible outliers associated with limited number of male subjects in each of studied groups (Figure 1). Taking all things into account, we decided to evaluate miRNAs expression changes only in female VAT samples.

### 3.4. The Set of Similarly Upregulated miRNAs Is Shared by T2DM and IFG Female Patients in VAT

**Figure 4:**
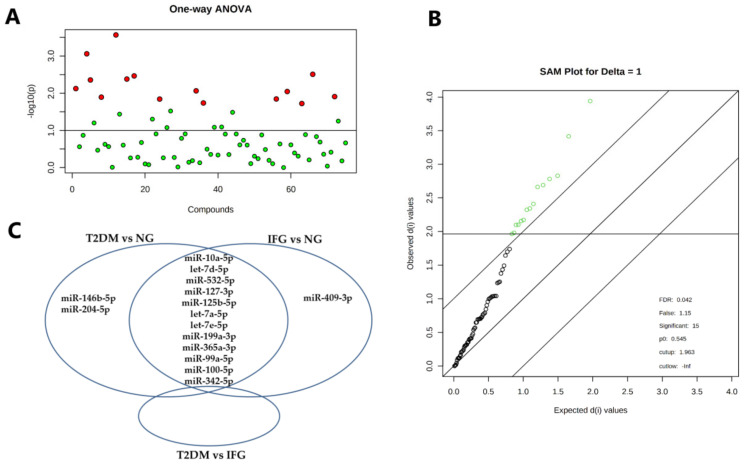
Results of one-way ANOVA and SAM indicating significantly changed miRNAs among three studied groups (T2DM, IFG, NG) of women (N = 26). (**A**) 15 molecules marked red were DE with FDR < 0.1 according to one-way ANOVA. Only two top miRNAs (miR-10a-5p and let-7d-5p) met the criteria of FDR < 0.05. Graph was generated by Metaboanalyst 4.0. Red and green dots indicated significantly and non-significantly changed miRNAs, respectively. (**B**) Graphical presentation of SAM results with FDR < 0.042 by Metaboanalyst 4.0. Green and black dots indicated significantly and non-significantly changed miRNAs, respectively. (**C**) Venn diagram of significantly upregulated miRNAs (FDR < 0.1), as indicated by one-way ANOVA and Tukey’s HSD post hoc test (*p* ≤ 0.05).

**Figure 5:**
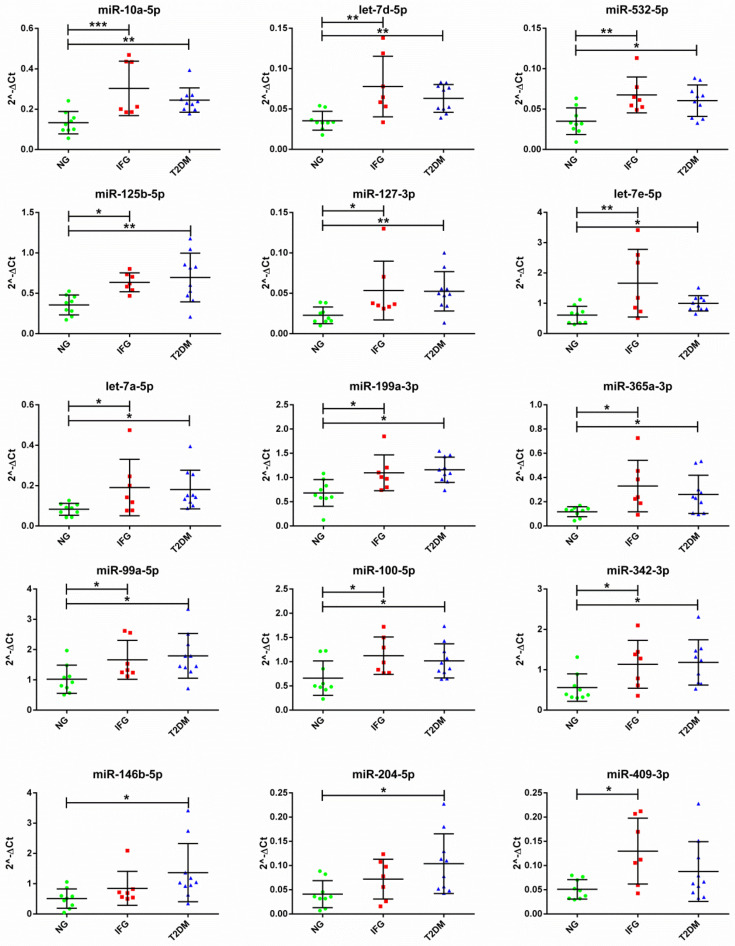
The expression profiles of 15 significantly changed miRNAs (FDR < 0.1) among T2DM, IFG and NG females (N = 26), based on one-way ANOVA and Tukey’s HSD post hoc test. Data was presented as dot plots with mean ± SD. Expression of the miRNAs was normalized to expression levels of miR-93-5p and miR-17-5p. Green dots, red squares and blue triangles represent results obtained for NG, IFG and T2DM females, respectively. (*) *p* ≤ 0.05; (**) *p* ≤ 0.01; (***) *p* ≤ 0.001.

**Figure A6:**
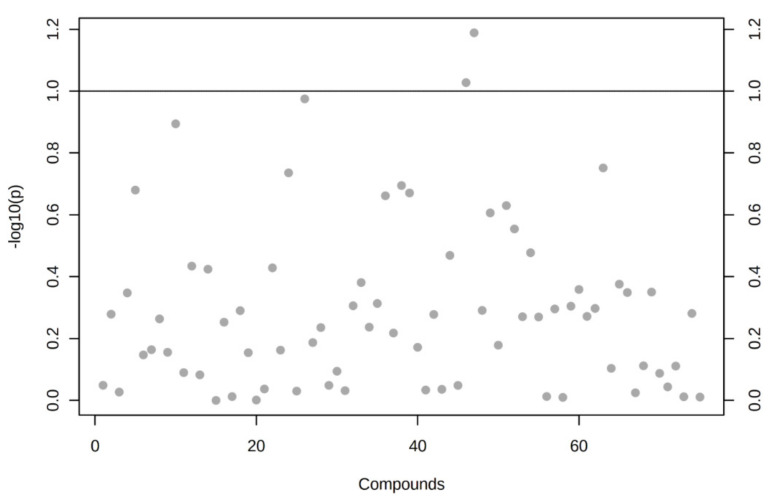
Graphical presentation of t-test analysis (FDR < 0.1) performed between T2DM and IFG female study subjects (N = 26) by Metaboanalyst 4.0. No significantly changed miRNAs were reported.

FDR adjustment (0.1) indicated that expression of 15 miRNAs was significantly changed among female study subjects (T2DM, IFG, NG) according to both one-way ANOVA and SAM (Figure 4A,B, Table 3). Importantly, only miR-10a-5p and let-7d-5p were reported to be upregulated with FDR < 0.05 by both analyses. Levels of miR-204-5p and miR-146b-5p were increased only in VAT of T2DM female subjects as compared to NG ones, while miR-409-3p was the only miRNA which increased solely in IFG subjects while comparing to NG ones (Figure 4C and Figure 5). None of tested miRNAs was changed among T2DM and IFG females according to one-way ANOVA (Figure 4C) and t-test (Figure A6). Further, 12 of the most DE miRNAs were similarly upregulated in VAT of both T2DM and IFG female subjects as compared to NG subjects (Figure 4C and Figure 5).

### 3.5. The Expression of Majority of Differentially Expressed miRNAs Is Significantly Correlated with Important Biochemical and Anthropometric Parameters in VAT of T2DM, IFG and NG Females

#### Spearman’s Rank Correlations among Differentially Expressed miRNAs and Biochemical and Anthropometric Parameters

**Figure 6:**
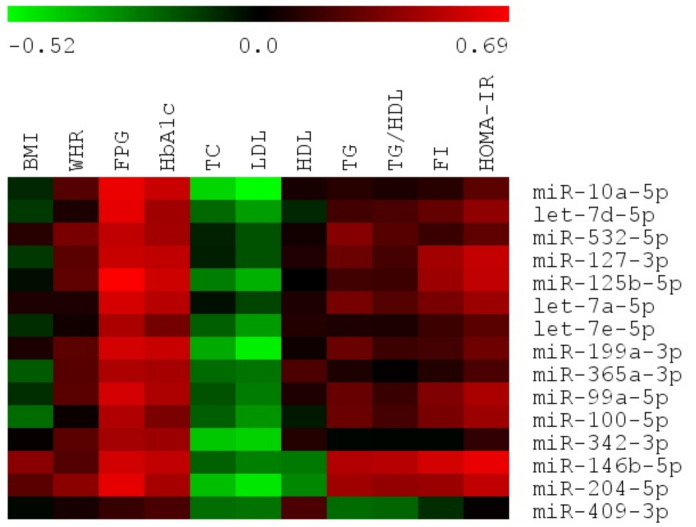
Heatmap of Spearman rank correlation coefficients calculated among expression levels of 15 DE miRNAs and anthropometric and biochemical parameters for female study subjects.

We also performed Spearman rank univariate correlation analyses with FDR correction (0.1) to evaluate the relationship among expression of DE miRNAs and important biochemical and anthropometric parameters in VAT of female study subjects (Figure 6, Table S4). Regarding anthropometric parameters, no significant correlations with WHR and BMI were found.

We found positive correlation between FPG and majority of DE miRNAs, apart from miR-342-3p and miR-409-3p, with highest values reached by miR-125b-5p, miR-10a-5p, let-7d-5d and miR-204-5p (adjusted *p* < 0.03). However, HbA1c levels were positively correlated only with miR-125b-5p, miR-10a-5p, miR-199a-3p, miR-146b-5p, miR-127-3p, let-7a-5p, miR-99a-5p, and miR-204-5p (adjusted *p* < 0.1).

Considering other parameters, we observed negative moderate correlation between LDL and miR-10a-5p, miR-199a-3p, miR-204-5p (r ≈ −0.5, adjusted *p* < 0.1). Exclusively miR-146b-5p expressed positive correlation with TG and IR indicator, TG/HDL (r ≈ 0.49, adjusted *p* < 0.08).

Spearman rank correlation among expression of miRNAs and FI and HOMA-IR needed additional exclusion of T2DM patients receiving insulin or sulfonylurea derivatives due to its direct impact on FI levels. After exclusion of six female T2DM patients, we observed positive correlation between HOMA-IR and miR-146b-5p, miR-204-5p, miR-127-3p, miR-125b-5p (adjusted *p* < 0.01). We also found positive relationship between FI and miR-146b-5p (adjusted *p* < 0.07).

Importantly, miR-409-3p was the only molecule which lacked significant correlative relationship with any parameter of adiposity (BMI, WHR) as well as glucose and lipid metabolism.

### 3.6. The Expression Levels of the Set of miRNAs Distinguish NG Female Subjects from T2DM and IFG Female Patients

#### 3.6.1. Univariate Receiving Operating Characteristic (ROC) Curve Analysis

**Figure 7:**
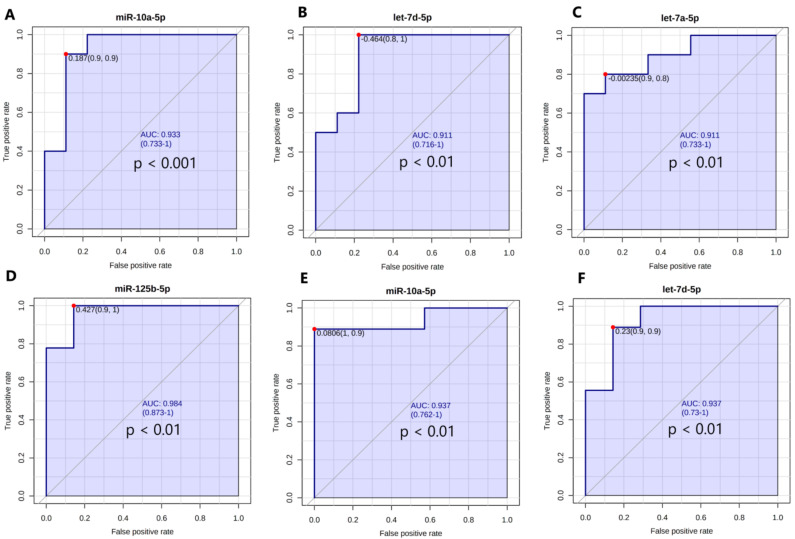
Biomarker analyses indicating 3 top miRNAs as potential VAT-specific markers of T2DM (T2DM vs. NG subjects) (**A**–**C**) as well as IFG (IFG vs. NG subjects) (**D**–**F**) in female subjects (N = 26) ranked according to area under ROC curve (AUROC). Optimal cutoff was calculated according to “closest to top—left corner method”. (**A**) miR-10a-5p, (**B**) let-7d-5p, (**C**) let-7a-5p, (**D**) miR-125b-5p, (**E**) miR-10a-5p, (**F**) let-7d-5p. Graphs were generated by Metaboanalyst 4.0.

In order to evaluate whether miRNAs could be considered as VAT-specific biomarkers of IFG or T2DM, we performed univariate ROC analyses. Interestingly, we found no miRNAs characterized by AUROC with *p* < 0.05 while investigating T2DM and IFG female patients. However, our analyses indicated that AUROCs calculated for all DE miRNAs apart from miR-409-3p reached statistical significance for T2DM and NG female subjects, while miR-10a-5p, let-7d-5p and let-7a-5p obtained top results (Figure 7A–C). We also observed that AUROCs of all DE miRNAs apart from miR-146b-5p and miR-204-5p reached statistical significance for IFG and NG female subjects, with miR-125b, miR-10a-5p, and let-7d-5p receiving the highest values (Figure 7D–F). The rest of values was presented in Table S5. Obtained data indicates that 15 DE miRNAs may be regarded as potential VAT-specific biomarkers of IFG and/or T2DM.

#### 3.6.2. Sparse Partial Least Squares-Discriminant Analysis (sPLS-DA)

**Figure 8:**
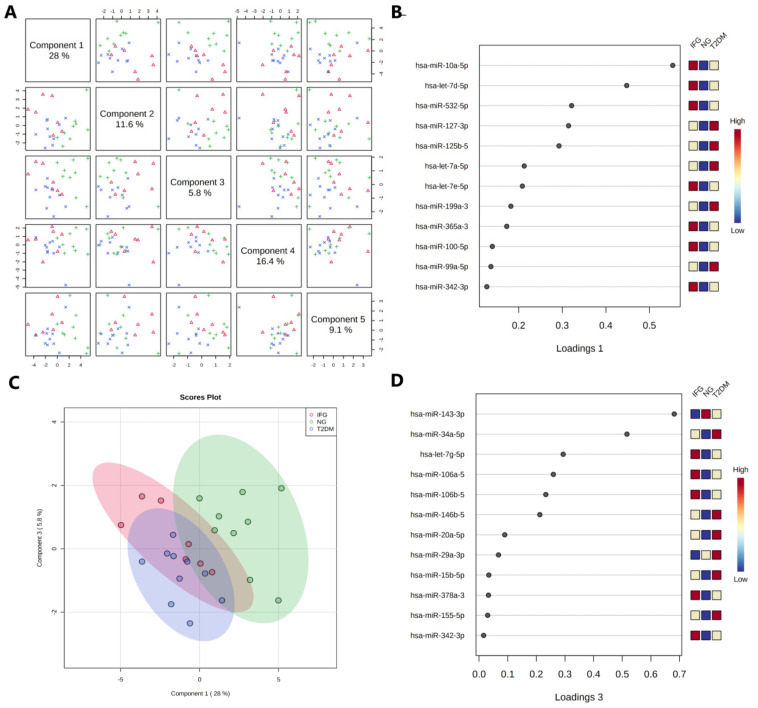
5-component sPLS-DA with LOOCV validation method performed for T2DM, IFG, NG female study subjects (N = 26). (**A**) Pairwise score plots overview with explained variance for each component. (**B**) Loading plot presenting miRNAs selected by sPLS-DA model for component 1. miRNAs were ranked according to their absolute values (**C**) Scores plot between components 1 and 3 with 95% confidence region. (**D**) Loading plot presenting miRNAs selected by sPLD-DA model for component 3. miRNAs were ranked according to their absolute values.

We performed sPLS-DA to (i) find out whether there exists a model enabling separation of female study participants while taking into account their clinical classification and miRNAs expression changes, and (ii) to confirm the selection of potential biologically relevant molecules (Figure 4C and Figure 5, Figure 6 and Figure 7). The analysis suggested that NG subjects can be successfully classified as substantially different group based on expression profile of miRNAs from component 1 and 3 of sPLS-DA model (Figure 8A–D). Interestingly, all 12 miRNAs belonging to component 1 (Figure 8B), a one explaining most of the expression data variance (28%), were DE and shared among IFG and T2DM female patients. The same conclusion could be also drawn from Figure 8C, showing scores plot indicating some dose of similarity among IFG and T2DM female patients.

#### 3.6.3. Euclidean Hierarchical Clustering

**Figure 9:**
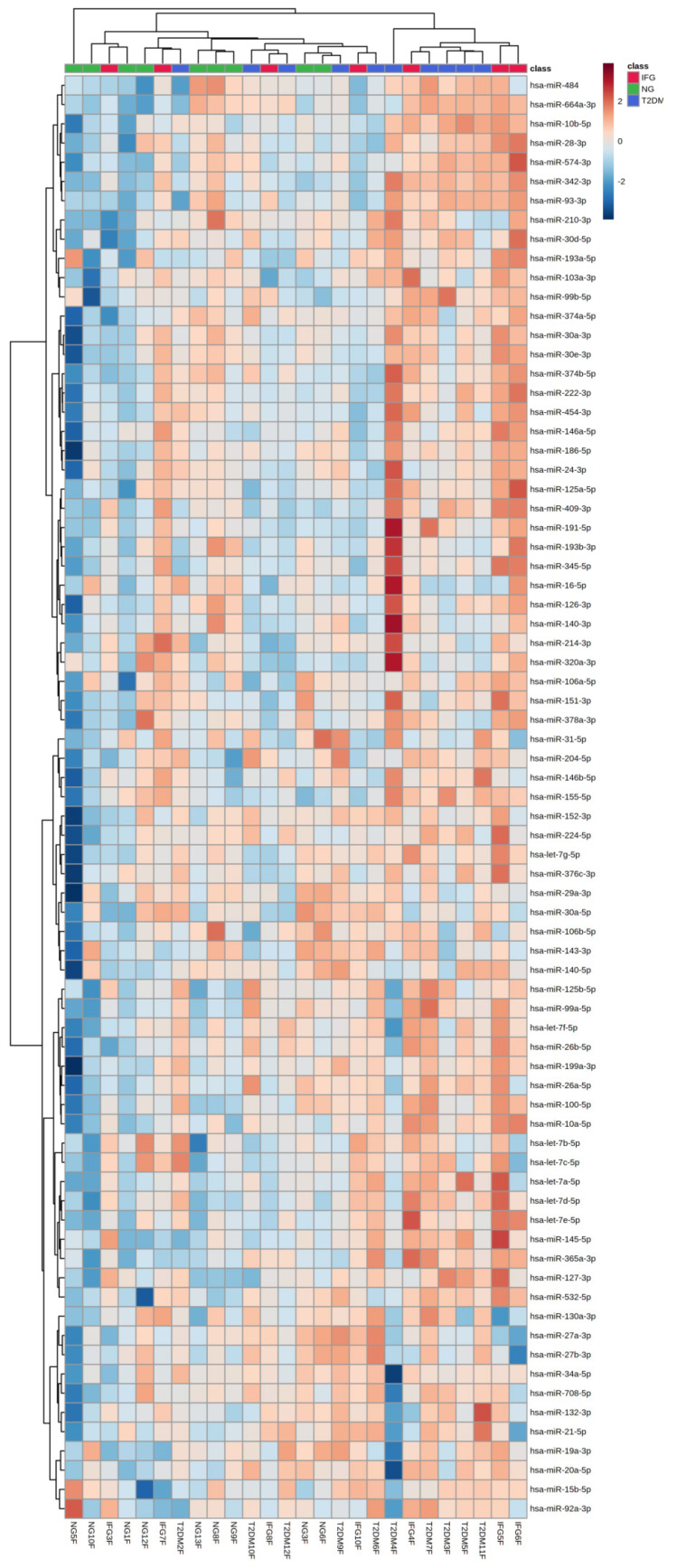
Euclidean hierarchical clustering among each female NG, IFG, T2DM subject (N = 26) and all tested miRNAs.

Euclidean hierarchical clustering performed for each female subject showed that only 8 females with either IFG or T2DM could be distinguished from other ones based on expression profile of 75 miRNAs in VAT (right cluster) (Figure 9). Moreover, clustering analysis indicated close relationship between levels of miR-204-5p and miR-146b-5p. It also determined top 11 DE miRNAs (miR-10a-5p, let-7d-5p, miR-532-5p, miR-127-3p, miR-125b-5p, let-7a-5p, let-7e-5p, miR-199a-3p, miR-365a-3p, miR-99a-5p, miR-100-5p) to form one cluster, in contrast to miR-342-3p. In overall, results suggested that top 11 DE miRNAs may be regulated in a common way and by a common factor, possibly, HG. Presented data also appears to indicate that there could be more miRNAs shared among IFG and T2DM groups, yet, not significantly changed in our limited population of women (Figure 9).

### 3.7. The Set of 11 Top Differentially Expressed miRNAs in VAT of Both T2DM and IFG Female Patients While Compared to NG Subjects is Functionally Annotated to Signaling Pathways Associated with Inflammation, Oxidative Stress and Insulin Signaling

Taking into account results of ANOVA, ROC curves, sPLS-DA modeling, Spearman’s correlations, and Euclidean hierarchical clustering, we decided to explore possible biological significance of top 11 DE miRNAs in VAT of both IFG and T2DM female patients while comparing to NG subjects via in silico analyses. The similar expression pattern of those miRNAs and their high discriminative potential suggest that they may act together to regulate similar target genes, signaling pathways or even biological phenomena in VAT of hyperglycemic patients.

Functional annotation performed by miRSystem for top 11 DE miRNAs indicated their involvement in DNA damage (ATR signaling pathway), IR (RhoA activity and GPCRs) and inflammation (Table 4). The latter was broadly represented by records associated with cytokines and their molecular signaling (JAK/STAT) and chemokines (e.g., NKT pathway).

**Figure A7:**
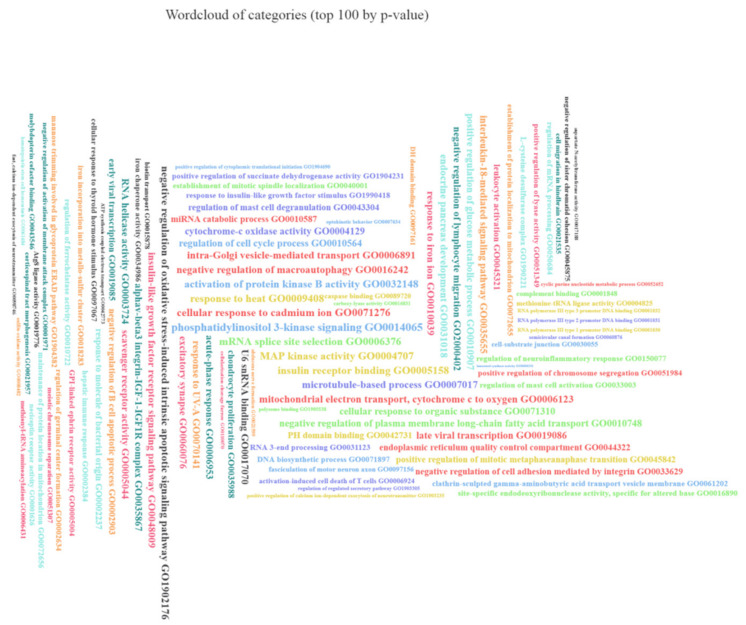
Functional annotations derived over miRTarBase (Gene Ontology) generated by miEAA for 11 significantly upregulated miRNAs shared among T2DM and IFG female subjects as compared to NG female subjects. GO–gene ontology.

**Figure A8:**
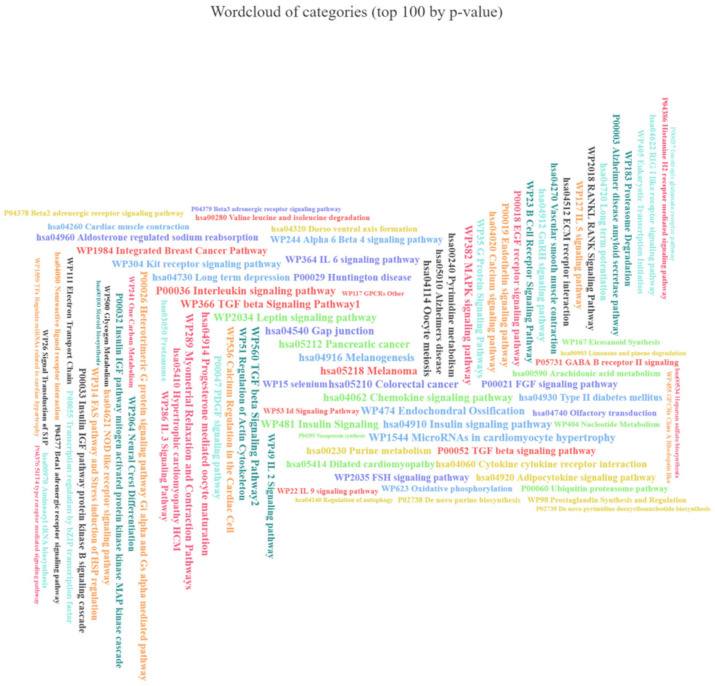
Results of enrichment analysis by miEAA for signaling pathways (miRWalk) generated for 11 significantly upregulated miRNAs shared among T2DM and IFG female subjects while compared to NG female subjects. WP (wikipathways), P (Panther Classification system), hsa (KEGG pathways).

Results provided by miEAA overrepresentation analyses also indicated phenomena related to inflammation with records regarding chemokines, cytokines, interleukins (including well-known markers of T2DM–IL-6 and IL-18), RANKL signaling, lymphocytes activation and migration (Figure A7 and Figure A8). miEAA also showed terms typically associated with VAT such as adipocytokine signaling, including leptin. The association with oxidative stress was also substantially indicated (negative regulation of oxidative stress-induced intrinsic apoptotic signaling pathway, cytochrome-c oxidase activity, mitochondrial electron transport, cytochrome c to oxygen, electron transport chain, oxidative phosphorylation, FAS pathway and stress induction of HSP regulation). Further, there were also records associated with glucose metabolism (type 2 diabetes, positive regulation of glucose metabolic process, signaling of insulin via INSR, IGF1R, PKB, Pi3K, and MAPK).

Altogether, in silico analyses indicated association among 11 selected miRNAs and oxidative stress, inflammation and glucose metabolism. From the clinical point of view, this could potentially suggest relationship with HG-evoked effects e.g., low-grade chronic inflammation, which demands thorough experimental confirmation.

## 4. Discussion

In the current study, we evaluated the impact of HG via measuring the relative expression of 75 miRNAs in VAT of male and female NG, IFG and T2DM subjects. To our best knowledge, this is the first study evaluating numerous miRNAs in VAT of male and female IFG subjects. Herein, we demonstrated that expression of 13 miRNAs was upregulated in VAT of IFG and/or T2DM patients as compared to NG ones in samples of 38 subjects, yet not after FDR correction. Our analyses also indicated that expression of miRNAs in VAT may be sex-dependent. Expression profile of numerous tested miRNAs appeared to be more similar among IFG and T2DM females, while in males, we noticed clustering among IFG and NG subjects. However, this observation should be taken with great caution as male study group was highly limited making whole analysis nonresistant to outliers. Indeed, expression of miRNAs was determined as sex-biased in human subjects [25]. In line with our data, previous observations confirmed that miRNAs expression in AT is affected by sex [26,27]. For instance, it was revealed that not only high-fat diet but also sex chromosome complement and gonadal hormones contribute to changes in mice miRNAs expression profile [27]. However, as the youngest woman in our study was 53 years old, we could expect that majority of female study participants were in postmenopausal period. Therefore, there are low chances of vast hormonal-based impact on observed miRNAs expression changes, while genetic and other factors cannot be excluded.

Next, we analyzed miRNAs expression solely in female VAT samples. We found 15 upregulated miRNAs in VAT of NG, IFG and T2DM females, while 12 top DE miRNAs were similarly increased in females with IFG and T2DM in comparison to NG subjects. However, statistical analyses make us speculate about strong interrelationship of 11 out of 12 similarly expressed miRNAs, further suggesting that those miRNAs could be regulated by a common factor, such as HG, and affect similar downstream signaling pathways plausibly associated with some clinical outcomes observed in hyperglycemic patients. As expected, in silico analyses concordantly indicated association of those 11 miRNAs with inflammation, oxidative stress, DNA damage and insulin signaling. Strikingly, Euclidean hierarchical clustering suggested that there could be far more miRNAs, yet not significantly changed in our relatively small female study group, that could be similarly regulated among IFG and T2DM subjects in comparison to NG ones. This may imply that expression of some miRNAs is sensitive to even slightly raised blood glucose levels, thus possibly participating in initiation and progression of low-grade chronic inflammation, HG-associated oxidative stress and reduction of insulin sensitivity observed both in IFG and T2DM patients. Hypothetically, such a similarity of miRNAs expression profiles may be also associated with some early compensative mechanism.

Upregulation of top DE miRNAs in VAT of hyperglycemic patients stays in line with studies evaluating expression changes of miR-125b-5p, miR-199a-3p, let-7d-5p and let-7e-5p upon HG or in serum and tissues of T2DM patients or animal models of diabetes [28,29,30,31]. miR-365a-3p was found to be most highly correlated, along with miR-125b-5p, with HbA1c in serum of type 1 diabetic subjects [32], similarly to our data. Some miRNAs such as miR-342-3p, miR-10a-5p and miR-146b-5p were also found to be increased in obese AT, in contrast to let-7a-5p [33,34,35]. However, miR-99a-5p and miR-100-5p were reduced in VAT of diabetic mice or subjects [36,37].

Our functional analysis was limited to in silico tools which mainly showed association with inflammation, oxidative stress and insulin signaling. Literature data seems to further confirm these observations, indicating involvement of miR-10a-5p, miR-127-3p, miR-125b-5p along with aforementioned miR-155-5p in macrophages polarization [38,39]. miR-127-3p was also suggested to regulate macrophages polarization-dependent secretion of proinflammatory cytokines by activating JNK pathway in lungs [40]. Indeed, the character of AT macrophages is easily changeable upon environmental stimuli such as high-fat diet, being associated with low-grade chronic inflammation [41]. Moreover, miR-342-3p exhibited 6-fold increase in inflamed adipocytes due to treatment with macrophages LPS-conditioned medium [42]. miR-199a-3p level increased upon exposure to TNF-α, IL-6 and free fatty acids in mature HPA-v adipocytes [43].

Some data also links expression changes of selected miRNAs with insulin sensitivity. For instance, miR-532-5p was associated with IR in females via regression analysis [44] as well as confirmed to target FoxO1 [45]. Further, let-7 family is a well-known regulator of glucose homeostasis and IR [46]. miR-99a-5p and miR-100-5p were confirmed to be direct regulators of AKT1, mTOR and IGF-1R [37,47,48,49], while let-7e-5p was proved to target IRS2 [31].

Inflammation and insulin sensitivity are inseparably connected with oxidative stress. In our recent review, we presented studies showing the impact of metabolic syndrome-related oxidative stress on numerous miRNAs, highlighting their impact on both prooxidant and antioxidant genes [9]. Although the knowledge about this issue is still in its infancy, transplantation of brown AT evoked increase of miR-99a-5p, which was confirmed to target NOX4, a prooxidant gene, and therefore, ameliorate diabetic phenotype in mice [50]. It is plausible that similarly upregulated miRNAs in VAT of hyperglycemic patients could affect genes capable of profound downstream regulation of prooxidant and antioxidant genes as evidenced for aforementioned FoxO1 (miR-532-5p), leading to burst of oxidative stress. Moreover, miR-125b-5p is predicted to target SIRT1 (a gene critical for oxidative defense), while being negatively correlated with its confirmed target, SIRT7, in VAT of obese Polish subjects [51].

miR-146b-5p and miR-204-5p were increased only in VAT of T2DM patients comparing to NG. In line with our data, miR-146b-5p was upregulated in VAT and SAT of obese subjects [52], while miR-204-5p was increased in VAT of T2DM patients with obesity, HG-treated 3T3-L1 mice adipocytes and AT of mice model of obesity [28,53]. Correlation analysis indicated their association with parameters of glucose metabolism and insulin resistance, thus further suggesting their role in progression of IR. Consistently, miR-146b-5p was earlier confirmed to indirectly target GLUT4 and directly target IRS1 in porcine primary adipocytes [54]. Both miRNAs were also proved to target SIRT1, a gene critical for adipogenesis, lipid and glucose metabolism, including IR [52,55]. For instance, miR-204-5p/SIRT1/AMPK/ACC axis was affected in AT of diabetic mice [56]. Considering phenomena related to inflammation, inhibition of miR-204-5p in mice model led to reduction of HG—associated inflammation and IR via promotion of SIRT1/GLUT4/PPARγ/AKT signaling [53]. Other studies showed miR-146b-5p upregulation in response to IL-6 and TNF-α in mature visceral adipocytes and its positive impact on expression of IL-6 in endothelial cells [57,58]. Altogether, data suggests that upregulation of miR-204-5p and miR-146b-5p may affect insulin sensitivity and promote inflammation in VAT of T2DM females.

The last molecule, miR-409-3p, was upregulated only in VAT of IFG females and uncorrelated with any biochemical and anthropometric parameter. Thus, miR-409-3p may be transiently changed in response to increased blood glucose levels in VAT. Its role may be connected with regulation of insulin signaling (Akt1, Pdk1) and antioxidant defense genes (e.g., Sod1) [59,60,61]. Considering inflammation, miR-409-3p was confirmed to increase production of inflammatory cytokines via regulating SOCS3/STAT3 pathway [62]. Although being still unexamined in white AT, its downregulation was observed in serum of T2DM patients [63].

Although performed on the limited number of subjects, our study investigated the expression of numerous miRNAs in relatively infrequently examined material and in subjects of both sexes. Further, obtained data highlighted possible sex-specific regulation of miRNAs expression in VAT. While VAT-specific DE miRNAs could be characterized by the limited diagnostic potential, our results may stimulate novel research oriented on the impact of VAT-specific exosomal miRNAs on the whole body glucose tolerance. Moreover, as only some of revealed miRNAs have been connected with T2DM, including studies on serum samples, our results may contribute to elucidation of novel serum-specific biomarkers of prediabetes and diabetes. The hypothesis testing whether significantly changed miRNAs could become useful in predicting the progression from normoglycemic to hyperglycemic state would be also valuable. Finally, the evaluation of the relationship among DE miRNAs and prooxidant and antioxidant genes or their molecular upstream regulators could serve as an interesting topic of novel basic research.

## 5. Conclusions

Obtained results suggest that expression of some miRNAs in VAT may be sex-specific, yet, it should be highlighted that our study was performed on the limited number of male subjects, making our analysis nonresistant to possible outliers. Furthermore, 15 out of 75 tested miRNAs were upregulated in VAT of female patients with T2DM and/or IFG as compared to NG subjects. Expression changes of some of those miRNAs have not been revealed in VAT of T2DM and IFG patients so far. Moreover, their expression levels were uncorrelated with adiposity indicators (BMI and WHR), while substantial number of DE miRNAs showed positive correlation with FPG and HbA1c even after FDR correction. Functional enrichment analysis indicated that top 11 DE and similarly upregulated miRNAs in hyperglycemic patients were associated with regulation of oxidative stress, inflammation, and insulin signaling. Altogether, future studies are needed to confirm in silico-derived observations that upregulation of those 11 miRNAs may be connected with low-grade chronic inflammation and oxidative stress in VAT of hyperglycemic subjects.

## Figures and Tables

**Table 1:** Characteristics of study subjects. Data is presented as median with interquartile range, significance level for intergroup differences was calculated with *Kruskal-Wallis one-way analysis of variance (ANOVA).*

	Sub.	NG (9F/4M)	IFG (7F/5M)	T2DM (10F/3M)
**Age [years]**	A	66 (61, 71)	65 (61.5, 77)	68 (64, 72)
	F	66 (61, 69)	66 (63, 77)	67 (64, 70)
	M	65.5 (60, 71)	64 (60, 80)	72 (62, 77)
**FPG [mmol/L]**	A	5.06 (4.82, 5.14)	5.99 (5.95, 6.42) (**)	7.46 (7.07, 8.12) (***)
	F	5.09 (4.85, 5.15)	5.98 (5.93, 6.64)	7.4 (7.07, 8.12) (***)
	M	4.46 (3.65, 5.10)	6 (5.98, 6.13)	10.15 (5.58, 19.11) (*)
**FI [pmol/L] &**	A	32.4 (25.30, 58.70)	48.75 (38.2, 76.95)	80.4 (40.3, 136.1)
	F	37.3 (29.00, 72.00)	43.7 (36.10, 67.60)	103.1 (74.7, 136.1)
	M	24.75 (11.50, 32.30)	50.9 (46.60, 172.90)	-
**HOMA-IR &**	A	1.05 (0.79, 2.09)	1.89 (1.59, 3.12)	3.71 (1.72, 8.34)
	F	1.22 (0.95, 2.37)	1.63 (1.59, 2.57)	5.35 (3.37, 8.34) (*)
	M	0.74 (0.28, 1.05)	1.95 (1.83, 6.20)	-
**HbA1c [%]**	A	5.5 (5.4, 5.5)	5.8 (5.7, 5.9) (**)	6.7 (6, 7) (***), (#)
	F	5.5 (5.4, 5.6)	5.9 (5.7, 5.9)	6.45 (6, 7) (***)
	M	5.35 (5.1, 5.5)	5.8 (5.7, 5.8)	6.7 (5.9, 9.1) (**)
**TC [mmol/L]**	A	4.98 (4.27, 5.39)	3.49 (2.94, 3.93) (*)	4.72 (2.89, 5.38)
	F	4.98 (4.31, 5.39)	3.62 (3.27, 4.10)	3.98 (2.89, 5.38)
	M	4.06 (2.87, 5.66)	2.9 (2.20, 3.80)	4.72 (2.87, 5.39)
**LDL-C [mmol/L]**	A	2.95 (2.64, 3.75)	1.77 (1.24, 2.28) (*)	2.66 (1.2, 2.93)
	F	3.37 (2.68, 3.75)	2.02 (1.36, 2.50)	2.05 (0.85, 2.78)
	M	2.17 (1.27, 3.55)	1.31 (0.89, 2.20)	2.93 (1.22, 3.40)
**HDL-C [mmol/L]**	A	1.22 (1.11, 1.35)	1.11 (0.92, 1.36)	1.19 (1.07, 1.25)
	F	1.22 (1.11, 1.29)	1.15 (0.87, 1.60)	1.19 (1.07, 1.25)
	M	1.26 (1.07, 1.40)	1.06 (0.96, 1.16)	1.29 (0.67, 1.61)
**TG [mmol/L]**	A	1.08 (1.01, 1.49)	1.06 (0.71, 1.27)	1.53 (1.25, 2.2) (#)
	F	1.06 (1.01, 1.18)	1.11 (0.85, 1.63)	1.7 (1.25, 2.31) (*)
	M	1.56 (1.07, 1.66)	0.72 (0.69, 1.16)	1.43 (0.79, 1.86)
**TG/HDL**	A	2.14 (1.79, 2.57)	1.92 (1.33, 2.89)	2.93 (2.65, 4.27)
	F	2.01 (1.79, 2.17)	1.99 (1.34, 4.29)	3.21 (2.78, 4.27) (*)
	M	2.56 (1.91, 3.27)	1.84 (1.32, 2.51)	2.65 (1.40, 4.89)
**BMI [kg/m^2^]**	A	27.44 (25.95, 27.99)	27.83 (25.48, 31.02)	30.84 (27.34, 33.20)
	F	27.51 (27.01, 27.99)	28.84 (25.00, 32.72)	30.31 (27.34, 33.91)
	M	26.7 (25.84, 28.08)	26.12 (25.95, 30.86)	31.14 (24.88, 31.14)
**Body mass [kg]**	A	74 (70, 80)	77.5 (71, 91)	77 (72, 89)
	F	72 (69, 74)	73 (64, 88)	75.5 (70, 85)
	M	80 (76, 84)	80 (75, 96)	90 (77, 90)
**Height [m]**	A	1.64 (1.61, 1.7)	1.68 (1.59, 1.75)	1.6 (1.58, 1.63)
	F	1.64 (1.61, 1.64)	1.6 (1.58, 1.65)	1.6 (1.57, 1.62)
	M	1.72 (1.7, 1.75)	1.75 (1.7, 1.76)	1.7 (1.7, 1.76)
**WHR**	A	0.94 (0.86, 0.96)	0.93 (0.90, 0.96)	1.02 (0.92, 1.06)
	F	0.9 (0.85, 0.94)	0.92 (0.84, 0.95)	0.97 (0.95, 1.02)
	M	1.00 (0.95, 1.05)	0.96 (0.92, 0.97)	1.06 (1.04, 1.09)
**Waist circ. [cm]**	A	100 (89.0, 103.0)	97.5 (85.0, 105.0)	104 (99.0, 112.0)
	F	90 (88.0, 100.0)	95 (80.0, 105.0)	102.5 (98.0, 109.0)
	M	103.5 (102.5, 104.5)	100 (90.0, 105.0)	115 (102.0, 117.0)
**Hips circ. [cm]**	A	105 (98.0, 110.0)	102.5 (97.0, 112.0)	107 (104.0, 110.0)
	F	105 (104.0, 111.0)	102 (96.0, 114.0)	108.5 (104.0, 110.0)
	M	104 (97.5, 110)	103 (98.0, 110.0)	107 (98.0, 109.0)

List of abbreviations: Sub.—subjects, A—all study subjects (N = 38), F—females (N = 26), M—males (N = 12), NG—normoglycemic subjects, IFG—impaired fasting glucose subjects, T2DM—type 2 diabetic subjects, BMI—body mass index, WHR—waist to hip ratio, FPG—fasting plasma glucose, FI—fasting insulin, HOMA-IR—homeostasis model assessment for insulin resistance, HbA1c—glycated hemoglobin, TC—total cholesterol, LDL-C—low-density lipoprotein cholesterol, HDL-C—high-density lipoprotein cholesterol, TG—triglycerides, circ—circumference. (&)—Levels of FI and HbA1c were compared for patients untreated with drugs directly affecting fasting insulin levels (only 5 diabetic subjects—4 females and 1 male). Significant differences in comparison to NG group are marked by (*), while (#) denotes significant difference among T2DM and IFG groups. */# *p* ≤ 0.05; ** *p* ≤ 0.01; *** *p* ≤ 0.001.

**Table 2:** Significantly changed miRNAs indicated for intergroup comparisons (T2DM, IFG, NG) by one—way ANOVA (FDR < 0.2) and SAM (FDR < 0.1) for all study subjects (N = 38) along with Spearman’s correlation coefficients for FPG and HbA1c (*p* values after FDR correction were only shown).

miRNA	One-Way ANOVA		SAM	
	Raw *p*	FDR	Raw *p*	FDR
miR-127-3p	0.0056	0.1439	0.0033	0.0560
miR-125b-5p	0.0077	0.1439	0.0048	0.0560
let-7d-5p	0.0097	0.1439	0.0068	0.0560
miR-155-5p	0.0110	0.1439	0.0080	0.0560
miR-342-3p	0.0152	0.1439	0.0112	0.0560
miR-532-5p	0.0180	0.1439	0.0132	0.0560
miR-10a-5p	0.0180	0.1439	0.0132	0.0560
let-7a-5p	0.0186	0.1439	0.0139	0.0560
miR-99a-5p	0.0189	0.1439	0.0140	0.0560
miR-146b-5p	0.0192	0.1439	0.0144	0.0560
let-7e-5p	0.0211	0.1439	0.0155	0.0560
miR-409-3p	0.0256	0.1571	0.0185	0.0613
miR-199a-3p	0.0272	0.1571	0.02	0.0613

**Table 3:** Significantly changed miRNAs indicated for intergroup comparisons (T2DM, IFG, NG) by one-way ANOVA and SAM for female subjects (N = 26) with FDR < 0.1 for both analyses. SAM results were generated by Metaboanalyst 4.0.

miRNA	One-Way ANOVA		SAM	
	Raw *p* Value	FDR	Raw *p* Value	FDR
miR-10a-5p	0.0003	0.0204	1.333E-4	0.0054
let-7d-5p	0.0009	0.0328	6.666E-4	0.0136
miR-532-5p	0.0031	0.0552	0.0021	0.0254
miR-127-3p	0.0034	0.0552	0.0025	0.0254
miR-125b-5p	0.0042	0.0552	0.0036	0.0254
let-7a-5p	0.0044	0.0552	0.0037	0.0254
let-7e-5p	0.0075	0.0753	0.0060	0.0333
miR-199a-3p	0.0087	0.0753	0.0073	0.0333
miR-365a-3p	0.0090	0.0753	0.0073	0.0333
miR-99a-5p	0.0124	0.0831	0.0104	0.0377
miR-100-5p	0.0128	0.0831	0.0105	0.0377
miR-342-3p	0.0143	0.0831	0.0119	0.0377
miR-146b-5p	0.0144	0.0831	0.0120	0.0377
miR-204-5p	0.0183	0.0950	0.0149	0.0418
miR-409-3p	0.0190	0.0950	0.0153	0.0418

**Table 4:** Significant terms generated for enrichment analysis performed by miRSystem for top 11 upregulated miRNAs shared among T2DM and IFG female patients while compared to NG subjects. Provided terms were sorted by empirical *p* value and significantly changed according to both raw and empirical *p* values.

Category	Term	Raw *p* Value	Empirical *p* Value
KEGG	CYTOKINE-CYTOKINE_RECEPTOR_INTERACTION	1.01 × 10^−3^	3.03 × 10^−4^
REACTOME	CHEMOKINE_RECEPTORS_BIND_CHEMOKINES	3.72 × 10^−3^	8.25 × 10^−3^
REACTOME	CLASS_A_1_(RHODOPSIN-LIKE_RECEPTORS)	7.34 × 10^−3^	1.24 × 10^−2^
BIOCARTA	BIOCARTA_NKT_PATHWAY	1.12 × 10^−2^	1.24 × 10^−2^
KEGG	JAK-STAT_SIGNALING_PATHWAY	1.13 × 10^−2^	1.57 × 10^−2^
BIOCARTA	BIOCARTA_INFLAM_PATHWAY	1.20 × 10^−2^	1.73 × 10^−2^
REACTOME	GPCR_LIGAND_BINDING	7.99 × 10^−3^	2.19 × 10^−2^
PAD	ATR_SIGNALING_PATHWAY	2.07 × 10^−2^	2.75 × 10^−2^
PAD	REGULATION_OF_RHOA_ACTIVITY	2.77 × 10^−2^	2.89 × 10^−2^
REACTOME	PEPTIDE_LIGAND-BINDING_RECEPTORS	1.96 × 10^−2^	4.72 × 10^−2^

KEGG—Kyoto Encyclopedia of Genes and Genomes, PAD—Pathway Interaction Database.

## Data Availability

The data presented in this study are available on request from the corresponding author.

## Supplementary Materials

The following are available online at https://www.mdpi.com/2076-3921/10/1/101/s1, Figure S1. Data autoscaling (feature view) regarding all study subjects (N = 38) by Metaboanalyst. Density plots involved all features (miRNAs), while boxplots were presented for only top 50 molecules. Data was mean-centered, and divided by the standard deviation (SD) of each variable, Figure S2. Data autoscaling (sample view) regarding all study subjects (N = 38)) by Metaboanalyst. Density plots and boxplots involved all samples (N = 38). Data was mean-centered, and divided by the standard deviation (SD) of each variable. Study subjects were marked dependently on studied groups: diabetic patients (T2DMF (female)/T2DMM (male)), prediabetic patients (IFGF (female)/IFGM (male)), normoglycemic (NGF (female)/NGM (male)), Figure S3. Data autoscaling (feature view) regarding female study subjects (N = 26). Density plots involved all features (miRNAs), while boxplots were presented for only top 50 molecules. Data was mean-centered, and divided by the standard deviation (SD) of each variable, Figure S4. Data autoscaling (sample view) regarding female study subjects (N = 26). Density plots and boxplots involved all samples (N = 26). Data was mean-centered, and divided by the standard deviation (SD) of each variable. Study subjects were marked dependently on studied groups: diabetic females (T2DMF), prediabetic females (IFGF), normoglycemic females (NGF). Table S1. Full list of assay IDs with names of molecules, Table S2. Power calculations for all miRNAs for all study subjects (N = 38) and solely for female study participants (N = 26), Table S3. Spearman correlation coefficients for DE miRNA with FDR adjustment for male and female study participants (N = 38). Results for FI and HOMA-IR were obtained after exclusion of 8 subjects treated with drugs affecting fasting insulin, Table S4. Spearman correlation coefficients for DE miRNA with FDR adjustment for females study participants (N = 26). Results for FI and HOMA-IR were obtained after exclusion of 6 females treated with drugs affecting fasting insulin, Table S5. ROC curves values characteristics generated among IFG and NG female subjects and T2DM and NG females.

## References

[1] Grundy S.M. (2012). Pre-diabetes, metabolic syndrome, and cardiovascular risk. J. Am. Coll. Cardiol..

[2] Bansal N. (2015). Prediabetes diagnosis and treatment: A review. World J. Diabetes.

[3] Liu P.J., Ma F., Lou H.P., Chen Y. (2016). Visceral adiposity index is associated with pre-diabetes and type 2 diabetes mellitus in Chinese adults aged 20–50. Ann. Nutr. Metab..

[4] Kintscher U., Hartge M., Hess K., Foryst-Ludwig A., Clemenz M., Wabitsch M., Fischer-Posovszky P., Barth T.F., Dragun D., Skurk T. (2008). T-lymphocyte infiltration in visceral adipose tissue: A primary event in adipose tissue inflammation and the development of obesity-mediated insulin resistance. Arterioscler. Thromb. Vasc. Biol..

[5] Brahimaj A., Ligthart S., Ghanbari M., Ikram M.A., Hofman A., Franco O.H., Kavousi M., Dehghan A. (2017). Novel inflammatory markers for incident pre-diabetes and type 2 diabetes: The Rotterdam Study. Eur. J. Epidemiol..

[6] Samaras K., Botelho N.K., Chisholm D.J., Lord R.V. (2010). Subcutaneous and visceral adipose tissue gene expression of serum adipokines that predict type 2 diabetes. Obesity.

[7] Burgos-Morón E., Abad-Jiménez Z., de Marañón A.M., Iannantuoni F., Escribano-López I., López-Domènech S., Salom C., Jover A., Mora V., Roldan I. (2019). Relationship between oxidative stress, ER stress, and inflammation in type 2 diabetes: The battle continues. J. Clin. Med..

[8] Ighodaro O.M. (2018). Molecular pathways associated with oxidative stress in diabetes mellitus. Biomed. Pharmacother..

[9] Włodarski A., Strycharz J., Wróblewski A., Kasznicki J., Drzewoski J., Śliwińska A. (2020). The Role of microRNAs in Metabolic Syndrome-Related Oxidative Stress. Int. J. Mol. Sci..

[10] Ghasemi A., Hashemy S.I., Azimi-Nezhad M., Dehghani A., Saeidi J., Mohtashami M. (2019). The cross-talk between adipokines and miRNAs in health and obesity-mediated diseases. Clin. Chim. Acta.

[11] Lorente-Cebrián S., González-Muniesa P., Milagro F.I., Martínez J.A. (2019). MicroRNAs and other non-coding RNAs in adipose tissue and obesity: Emerging roles as biomarkers and therapeutic targets. Clin. Sci..

[12] Ying W., Riopel M., Bandyopadhyay G., Dong Y., Birmingham A., Seo J.B., Ofrecio J.M., Wollam J., Hernandez-Carretero A., Fu W. (2017). Adipose tissue macrophage-derived exosomal miRNAs can modulate in vivo and in vitro insulin sensitivity. Cell.

[13] Lopez Y.O.N., Garufi G., Seyhan A.A. (2017). Altered levels of circulating cytokines and microRNAs in lean and obese individuals with prediabetes and type 2 diabetes. Mol. Biosyst..

[14] Yan S., Wang T., Huang S., Di Y., Huang Y., Liu X., Luo Z., Han W., An B. (2016). Differential expression of microRNAs in plasma of patients with prediabetes and newly diagnosed type 2 diabetes. Acta Diabetol..

[15] Prabu P., Rome S., Sathishkumar C., Aravind S., Mahalingam B., Shanthirani C.S., Gastebois C., Villard A., Mohan V., Balasubramanyam M. (2015). Circulating miRNAs of ‘Asian Indian phenotype’identified in subjects with impaired glucose tolerance and patients with type 2 diabetes. PLoS ONE.

[16] American Diabetes Association (2020). 2. Classification and Diagnosis of Diabetes: Standards of Medical Care in Diabetes-2020. Diabetes Care.

[17] Wenclewska S., Szymczak-Pajor I., Drzewoski J., Bunk M., Śliwińska A. (2019). Vitamin D supplementation reduces both oxidative DNA damage and insulin resistance in the elderly with metabolic disorders. Int. J. Mol. Sci..

[18] Strycharz J., Świderska E., Wróblewski A., Podolska M., Czarny P., Szemraj J., Balcerczyk A., Drzewoski J., Kasznicki J., Śliwińska A. (2018). Hyperglycemia Affects miRNAs Expression Pattern during Adipogenesis of Human Visceral Adipocytes—Is Memorization Involved?. Nutrients.

[19] Świderska E., Podolska M., Strycharz J., Szwed M., Abramczyk H., Brożek-Płuska B., Wróblewski A., Szemraj J., Majsterek I., Drzewoski J. (2019). Hyperglycemia changes expression of key adipogenesis markers (C/EBPα and PPARγ) and morphology of differentiating Human visceral adipocytes. Nutrients.

[20] Pfaffl M.W., Tichopad A., Prgomet C., Neuvians T.P. (2004). Determination of stable housekeeping genes, differentially regulated target genes and sample integrity: BestKeeper–Excel-based tool using pair-wise correlations. Biotechnol. Lett..

[21] Kern F., Fehlmann T., Solomon J., Schwed L., Backes C., Meese E., Keller A. (2020). miEAA 2.0: Integrating multi-species microRNA enrichment analysis and workflow management systems. bioRxiv.

[22] Lu T.-P., Lee C.-Y., Tsai M.-H., Chiu Y.-C., Hsiao C.K., Lai L.-C., Chuang E.Y. (2012). miRSystem: An integrated system for characterizing enriched functions and pathways of microRNA targets. PLoS ONE.

[23] Howe E., Holton K., Nair S., Schlauch D., Sinha R., Quackenbush J. (2010). Mev: Multiexperiment viewer. Biomedical Informatics for Cancer Research.

[24] Pang Z., Chong J., Li S., Xia J. (2020). MetaboAnalystR 3.0: Toward an Optimized Workflow for Global Metabolomics. Metabolites.

[25] Guo L., Zhang Q., Ma X., Wang J., Liang T. (2017). miRNA and mRNA expression analysis reveals potential sex-biased miRNA expression. Sci. Rep..

[26] Mentzel C.M.J., Anthon C., Jacobsen M.J., Karlskov-Mortensen P., Bruun C.S., Jørgensen C.B., Gorodkin J., Cirera S., Fredholm M. (2015). Gender and obesity specific microRNA expression in adipose tissue from lean and obese pigs. PLoS ONE.

[27] Link J.C., Hasin-Brumshtein Y., Cantor R.M., Chen X., Arnold A.P., Lusis A.J., Reue K. (2017). Diet, gonadal sex, and sex chromosome complement influence white adipose tissue miRNA expression. BMC Genom..

[28] Brovkina O., Nikitin A., Khodyrev D., Shestakova E., Sklyanik I., Panevina A., Stafeev Y.S., Menshikov M., Kobelyatskaya A., Yurasov A. (2019). Role of microRNAs in the regulation of subcutaneous white adipose tissue in obese patients without type 2 diabetes. Front. Endocrinol..

[29] Li Y.B., Wu Q., Liu J., Fan Y.Z., Yu K.F., Cai Y. (2017). miR-199a-3p is involved in the pathogenesis and progression of diabetic neuropathy through downregulation of SerpinE2. Mol. Med. Rep..

[30] Jiang L.Q., Franck N., Egan B., Sjögren R.J., Katayama M., Duque-Guimaraes D., Arner P., Zierath J.R., Krook A. (2013). Autocrine role of interleukin-13 on skeletal muscle glucose metabolism in type 2 diabetic patients involves microRNA let-7. Am. J. Physiol. Endocrinol. Metab..

[31] Krause C., Geißler C., Tackenberg H., El Gammal A.T., Wolter S., Spranger J., Mann O., Lehnert H., Kirchner H. (2020). Multi-layered epigenetic regulation of IRS2 expression in the liver of obese individuals with type 2 diabetes. Diabetologia.

[32] Satake E., Pezzolesi M.G., Dom Z.I.M., Smiles A.M., Niewczas M.A., Krolewski A.S. (2018). Circulating miRNA profiles associated with hyperglycemia in patients with type 1 diabetes. Diabetes.

[33] Oger F., Gheeraert C., Mogilenko D., Benomar Y., Molendi-Coste O., Bouchaert E., Caron S., Dombrowicz D., Pattou F., Duez H. (2014). Cell-specific dysregulation of microRNA expression in obese white adipose tissue. J. Clin. Endocrinol. Metab..

[34] Chartoumpekis D.V., Zaravinos A., Ziros P.G., Iskrenova R.P., Psyrogiannis A.I., Kyriazopoulou V.E., Habeos I.G. (2012). Differential expression of microRNAs in adipose tissue after long-term high-fat diet-induced obesity in mice. PLoS ONE.

[35] Ortega F.J., Moreno-Navarrete J.M., Pardo G., Sabater M., Hummel M., Ferrer A., Rodriguez-Hermosa J.I., Ruiz B., Ricart W., Peral B. (2010). MiRNA expression profile of human subcutaneous adipose and during adipocyte differentiation. PLoS ONE.

[36] Jaiswal A., Reddy S.S., Maurya M., Maurya P., Barthwal M.K. (2019). MicroRNA-99a mimics inhibit M1 macrophage phenotype and adipose tissue inflammation by targeting TNFα. Cell. Mol. Immunol..

[37] Pek S.L.T., Sum C.F., Lin M.X., Cheng A.K.S., Wong M.T.K., Lim S.C., Tavintharan S. (2016). Circulating and visceral adipose miR-100 is down-regulated in patients with obesity and Type 2 diabetes. Mol. Cell. Endocrinol..

[38] Cho Y.K., Son Y., Kim S.-N., Song H.-D., Kim M., Park J.-H., Jung Y.-S., Ahn S.-Y., Saha A., Granneman J.G. (2019). MicroRNA-10a-5p regulates macrophage polarization and promotes therapeutic adipose tissue remodeling. Mol. Metab..

[39] Essandoh K., Li Y., Huo J., Fan G.-C. (2016). MiRNA-mediated macrophage polarization and its potential role in the regulation of inflammatory response. Shock Augusta Ga..

[40] Ying H., Kang Y., Zhang H., Zhao D., Xia J., Lu Z., Wang H., Xu F., Shi L. (2015). MiR-127 modulates macrophage polarization and promotes lung inflammation and injury by activating the JNK pathway. J. Immunol..

[41] Weisberg S.P., McCann D., Desai M., Rosenbaum M., Leibel R.L., Ferrante A.W. (2003). Obesity is associated with macrophage accumulation in adipose tissue. J. Clin. Investig..

[42] Ortega F.J., Moreno M., Mercader J.M., Moreno-Navarrete J.M., Fuentes-Batllevell N., Sabater M., Ricart W., Fernández-Real J.M. (2015). Inflammation triggers specific microRNA profiles in human adipocytes and macrophages and in their supernatants. Clin. Epigenetics.

[43] Gu N., You L., Shi C., Yang L., Pang L., Cui X., Ji C., Zheng W., Guo X. (2016). Expression of miR-199a-3p in human adipocytes is regulated by free fatty acids and adipokines. Mol. Med. Rep..

[44] Jones A., Danielson K.M., Benton M.C., Ziegler O., Shah R., Stubbs R.S., Das S., Macartney-Coxson D. (2017). miRNA signatures of insulin resistance in obesity. Obesity.

[45] Guo X., Wei S., Xu F., Cai X., Wang H., Ding R. (2020). MicroRNA-532-5p is implicated in the regulation of osteoporosis by forkhead box O1 and osteoblast differentiation. BMC Musculoskelet. Disord..

[46] Frost R.J., Olson E.N. (2011). Control of glucose homeostasis and insulin sensitivity by the Let-7 family of microRNAs. Proc. Natl. Acad. Sci. USA.

[47] Zhang Z.-W., Guo R.-W., Lv J.-L., Wang X.-M., Ye J.-S., Lu N.-H., Liang X., Yang L.-X. (2017). MicroRNA-99a inhibits insulin-induced proliferation, migration, dedifferentiation, and rapamycin resistance of vascular smooth muscle cells by inhibiting insulin-like growth factor-1 receptor and mammalian target of rapamycin. Biochem. Biophys. Res. Commun..

[48] Yu S.H., Zhang C.L., Dong F.S., Zhang Y.M. (2015). miR-99a suppresses the metastasis of human non-small cell lung cancer cells by targeting AKT1 signaling pathway. J. Cell. Biochem..

[49] Jin Y., Tymen S.D., Chen D., Fang Z.J., Zhao Y., Dragas D., Dai Y., Marucha P.T., Zhou X. (2013). MicroRNA-99 family targets AKT/mTOR signaling pathway in dermal wound healing. PLoS ONE.

[50] Li P., Fan C., Cai Y., Fang S., Zeng Y., Zhang Y., Lin X., Zhang H., Xue Y., Guan M. (2020). Transplantation of brown adipose tissue up-regulates miR-99a to ameliorate liver metabolic disorders in diabetic mice by targeting NOX4. Adipocyte.

[51] Kurylowicz A., Owczarz M., Polosak J., Jonas M., Lisik W., Jonas M., Chmura A., Puzianowska-Kuznicka M. (2016). SIRT1 and SIRT7 expression in adipose tissues of obese and normal-weight individuals is regulated by microRNAs but not by methylation status. Int. J. Obes..

[52] Chen L., Dai Y.-M., Ji C.-B., Yang L., Shi C.-M., Xu G.-F., Pang L.-X., Huang F.-Y., Zhang C.-M., Guo X.-R. (2014). MiR-146b is a regulator of human visceral preadipocyte proliferation and differentiation and its expression is altered in human obesity. Mol. Cell. Endocrinol..

[53] Zhang Y., Gu M., Ma Y., Peng Y. (2020). LncRNA TUG1 reduces inflammation and enhances insulin sensitivity in white adipose tissue by regulating miR-204/SIRT1 axis in obesity mice. Mol. Cell. Biochem..

[54] Zhu Y.-L., Chen T., Xiong J.-L., Wu D., Xi Q.-Y., Luo J.-Y., Sun J.-J., Zhang Y.-L. (2018). miR-146b inhibits glucose consumption by targeting IRS1 gene in porcine primary adipocytes. Int. J. Mol. Sci..

[55] Strycharz J., Rygielska Z., Swiderska E., Drzewoski J., Szemraj J., Szmigiero L., Sliwinska A. (2018). SIRT1 as a therapeutic target in diabetic complications. Curr. Med. Chem..

[56] Zhang Y., Ma Y., Gu M., Peng Y. (2020). lncRNA TUG1 promotes the brown remodeling of white adipose tissue by regulating miR-204-targeted SIRT1 in diabetic mice. Int. J. Mol. Med..

[57] Shi C., Zhu L., Chen X., Gu N., Chen L., Zhu L., Yang L., Pang L., Guo X., Ji C. (2014). IL-6 and TNF-α induced obesity-related inflammatory response through transcriptional regulation of miR-146b. J. Interferon Cytokine Res..

[58] Pfeiffer D., Roßmanith E., Lang I., Falkenhagen D. (2017). miR-146a, miR-146b, and miR-155 increase expression of IL-6 and IL-8 and support HSP10 in an in vitro sepsis model. PLoS ONE.

[59] Wang Y., He Y., Bai H., Dang Y., Gao J., Lv P. (2019). Phosphoinositide-dependent kinase 1–associated glycolysis is regulated by miR-409-3p in clear cell renal cell carcinoma. J. Cell. Biochem..

[60] Zhang G., Liu Z., Xu H., Yang Q. (2016). miR-409-3p suppresses breast cancer cell growth and invasion by targeting Akt1. Biochem. Biophys. Res. Commun..

[61] Liu S., Li B., Xu J., Hu S., Zhan N., Wang H., Gao C., Li J., Xu X. (2020). SOD1 Promotes Cell Proliferation and Metastasis in Non-small Cell Lung Cancer via an miR-409-3p/SOD1/SETDB1 Epigenetic Regulatory Feedforward Loop. Front. Cell Dev. Biol..

[62] Liu X., Zhou F., Yang Y., Wang W., Niu L., Zuo D., Li X., Hua H., Zhang B., Kou Y. (2019). MiR-409-3p and MiR-1896 co-operatively participate in IL-17-induced inflammatory cytokine production in astrocytes and pathogenesis of EAE mice via targeting SOCS3/STAT3 signaling. Glia.

[63] Yang Z.M., Chen L.H., Hong M., Chen Y.Y., Yang X.R., Tang S.M., Yuan Q.F., Chen W.W. (2017). Serum microRNA profiling and bioinformatics analysis of patients with type 2 diabetes mellitus in a Chinese population. Mol. Med. Rep..

