# Supplementary material for: Visceral Adipose Tissue of Prediabetic and Diabetic Females Shares a Set of Similarly Upregulated microRNAs Functionally Annotated to Inflammation, Oxidative Stress and Insulin Signaling
Source: Antioxidants (Basel). 2021 Jan 12;10(1):101. doi: 10.3390/antiox10010101 (PMC7828194; doi:10.3390/antiox10010101)
Supplement: Supplementary file 1 [file antioxidants-10-00101-s001.pdf]

Supplementary Materials

Table S1. Full list of assay IDs with names of molecules.

Assay ID	Molecule
hsa-let-7a-000377	hsa-let-7a-5p
hsa-let-7b-002619	hsa-let-7b-5p
hsa-let-7c-000379	hsa-let-7c-5p
hsa-let-7d-002283	hsa-let-7d-5p
hsa-let-7e-002406	hsa-let-7e-5p
hsa-let-7f-000382	hsa-let-7f-5p
hsa-let-7g-002282	hsa-let-7g-5p
hsa-miR-100-000437	hsa-miR-100-5p
hsa-miR-103-000439	hsa-miR-103a-3p
hsa-miR-106a-002169	hsa-miR-106a-5p
hsa-miR-106b-000442	hsa-miR-106b-5p
hsa-miR-10a-000387	hsa-miR-10a-5p
hsa-miR-10b-002218	hsa-miR-10b-5p
hsa-miR-125a-5p-002198	hsa-miR-125a-5p
hsa-miR-125b-000449	hsa-miR-125b-5p
hsa-miR-126-002228	hsa-miR-126-3p
hsa-miR-127-000452	hsa-miR-127-3p
hsa-miR-130a-000454	hsa-miR-130a-3p
hsa-miR-132-000457	hsa-miR-132-3p
hsa-miR-140-3p-002234	hsa-miR-140-3p
hsa-miR-143-002249	hsa-miR-143-3p
hsa-miR-145-002278	hsa-miR-145-5p
hsa-miR-146a-000468	hsa-miR-146a-5p
hsa-miR-146b-001097	hsa-miR-146b-5p
hsa-miR-151-3p-002254	hsa-miR-151-3p
hsa-miR-152-000475	hsa-miR-152-3p
hsa-miR-155-002623	hsa-miR-155-5p
hsa-miR-15b-000390	hsa-miR-15b-5p
hsa-miR-16-000391	hsa-miR-16-5p
hsa-miR-186-002285	hsa-miR-186-5p
hsa-miR-191-002299	hsa-miR-191-5p
hsa-miR-193a-5p-002281	hsa-miR-193a-5p
hsa-miR-193b-002367	hsa-miR-193b-3p
hsa-miR-199a-3p-002304	hsa-miR-199a-3p
hsa-miR-19a-000395	hsa-miR-19a-3p
hsa-miR-204-000508	hsa-miR-204-5p
hsa-miR-20a-000580	hsa-miR-20a-5p
hsa-miR-210-000512	hsa-miR-210-3p
hsa-miR-21-000397	hsa-miR-21-5p
hsa-miR-214-002306	hsa-miR-214-3p
hsa-miR-222-002276	hsa-miR-222-3p
hsa-miR-224-002099	hsa-miR-224-5p
hsa-miR-24-000402	hsa-miR-24-3p
hsa-miR-26a-000405	hsa-miR-26a-5p
hsa-miR-26b-000407	hsa-miR-26b-5p
hsa-miR-27a-000408	hsa-miR-27a-3p
hsa-miR-27b-000409	hsa-miR-27b-3p

hsa-miR-28-3p-002446	hsa-miR-28-3p
hsa-miR-29a-002112	hsa-miR-29a-3p
hsa-miR-30a-3p-000416	hsa-miR-30a-3p
hsa-miR-30a-5p-000417	hsa-miR-30a-5p
hsa-miR-30d-000420	hsa-miR-30d-5p
hsa-miR-30e-3p-000422	hsa-miR-30e-3p
hsa-miR-31-002279	hsa-miR-31-5p
hsa-miR-320-002277	hsa-miR-320a-3p
hsa-miR-342-3p-002260	hsa-miR-342-3p
hsa-miR-345-002186	hsa-miR-345-5p
hsa-miR-34a-000426	hsa-miR-34a-5p
hsa-miR-365-001020	hsa-miR-365a-3p
hsa-miR-374-000563	hsa-miR-374a-5p
hsa-miR-376c-002122	hsa-miR-376c-3p
hsa-miR-378-002243	hsa-miR-378a-5p
hsa-miR-409-3p-002332	hsa-miR-409-3p
hsa-miR-454-002323	hsa-miR-454-3p
hsa-miR-484-001821	hsa-miR-484
hsa-miR-532-001518	hsa-miR-532-5p
hsa-miR-574-3p-002349	hsa-miR-574-3p
hsa-miR-664-002897	hsa-miR-664a-3p
hsa-miR-708-002341	hsa-miR-708-5p
hsa-miR-92a-000431	hsa-miR-92a-3p
hsa-miR-93*-002139	hsa-miR-93-3p
hsa-miR-99a-000435	hsa-miR-99a-5p
hsa-miR-99b-000436	hsa-miR-99b-5p
mmu-miR-140-001187	hsa-miR-140-5p
mmu-miR-374-5p-001319	hsa-miR-374-5p
hsa-miR-596-001550	hsa-miR-596
RNU44-001094	RNU44
RNU48-001006	RNU48
U47-001223	U47
U6 snRNA-001973	U6
hsa-miR-4267-242640_mat	negative control

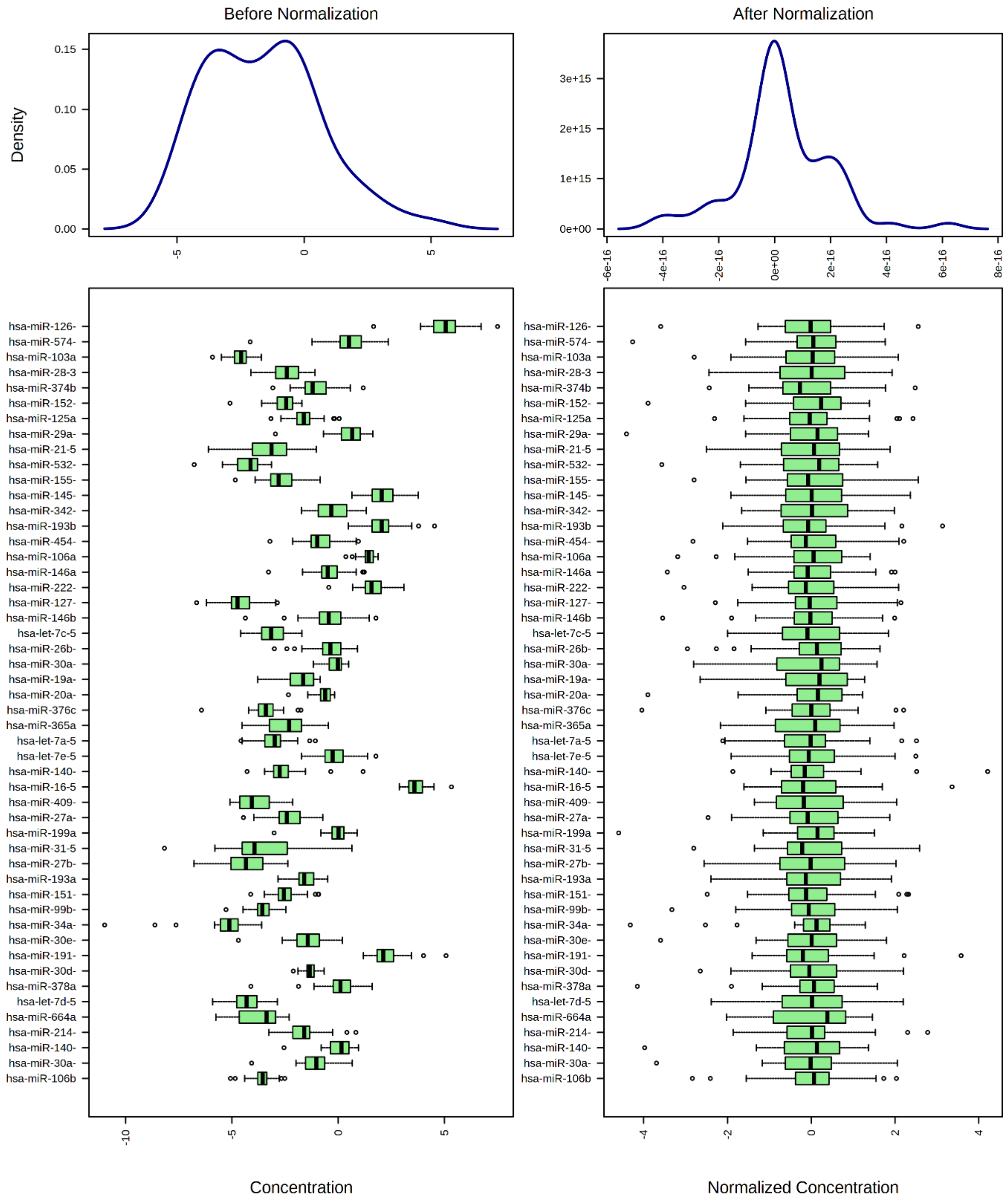

Figure S1. Data autoscaling (feature view) regarding all study subjects (N=38) by Metaboanalyst. Density plots involved all features (miRNAs), while boxplots were presented for only top 50 molecules. Data was mean-centered, and divided by the standard deviation (SD) of each variable.

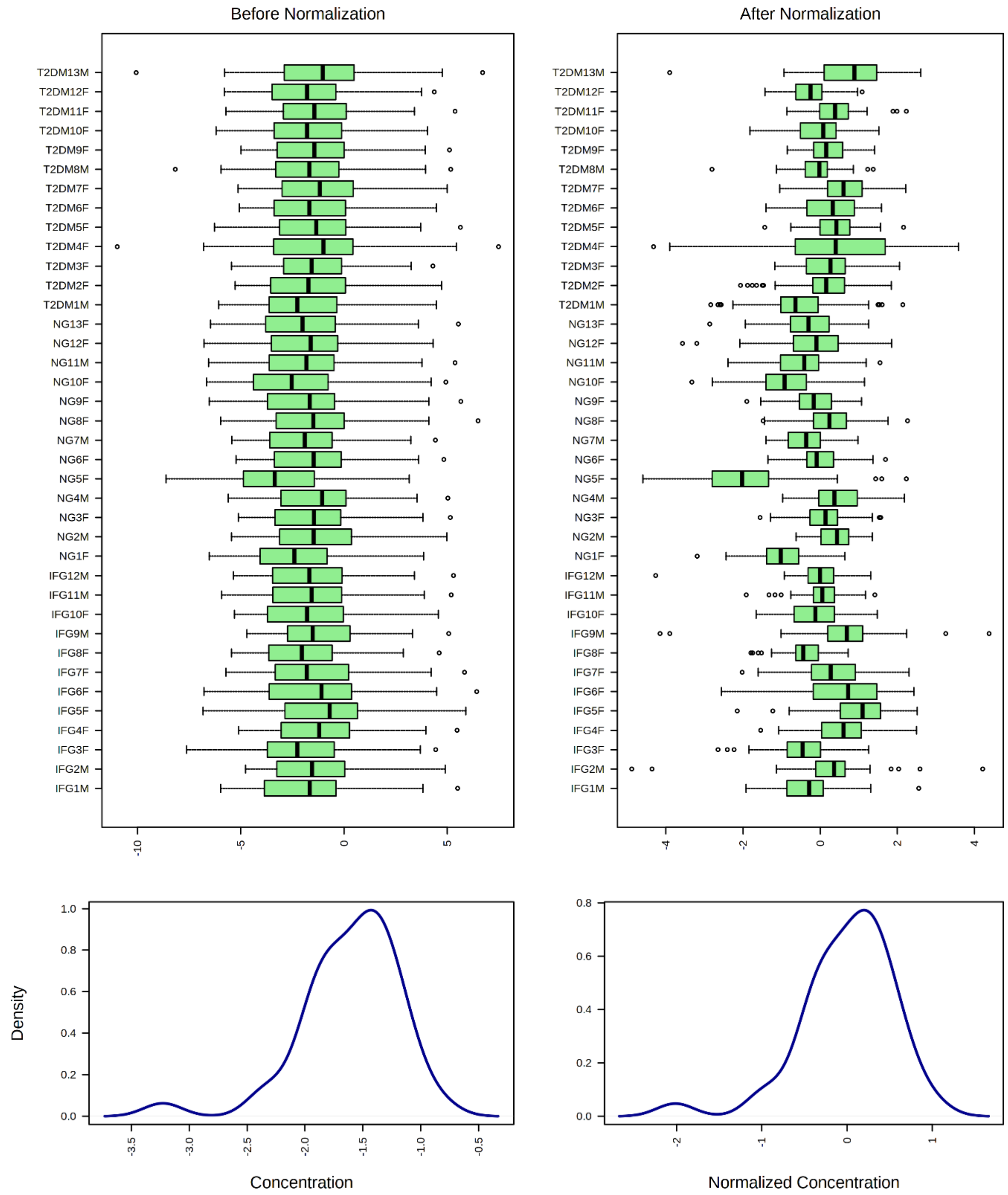

Figure S2. Data autoscaling (sample view) regarding all study subjects (N=38)) by Metaboanalyst. Density plots and box-plots involved all samples (N=38). Data was mean-centered, and divided by the standard deviation (SD) of each variable. Study subjects were marked dependently on studied groups : diabetic patients (T2DMF (female) / T2DMM (male)), pre-diabetic patients (IFGF (female) / IFGM (male)), normoglycemic (NGF (female) / NGM (male)).

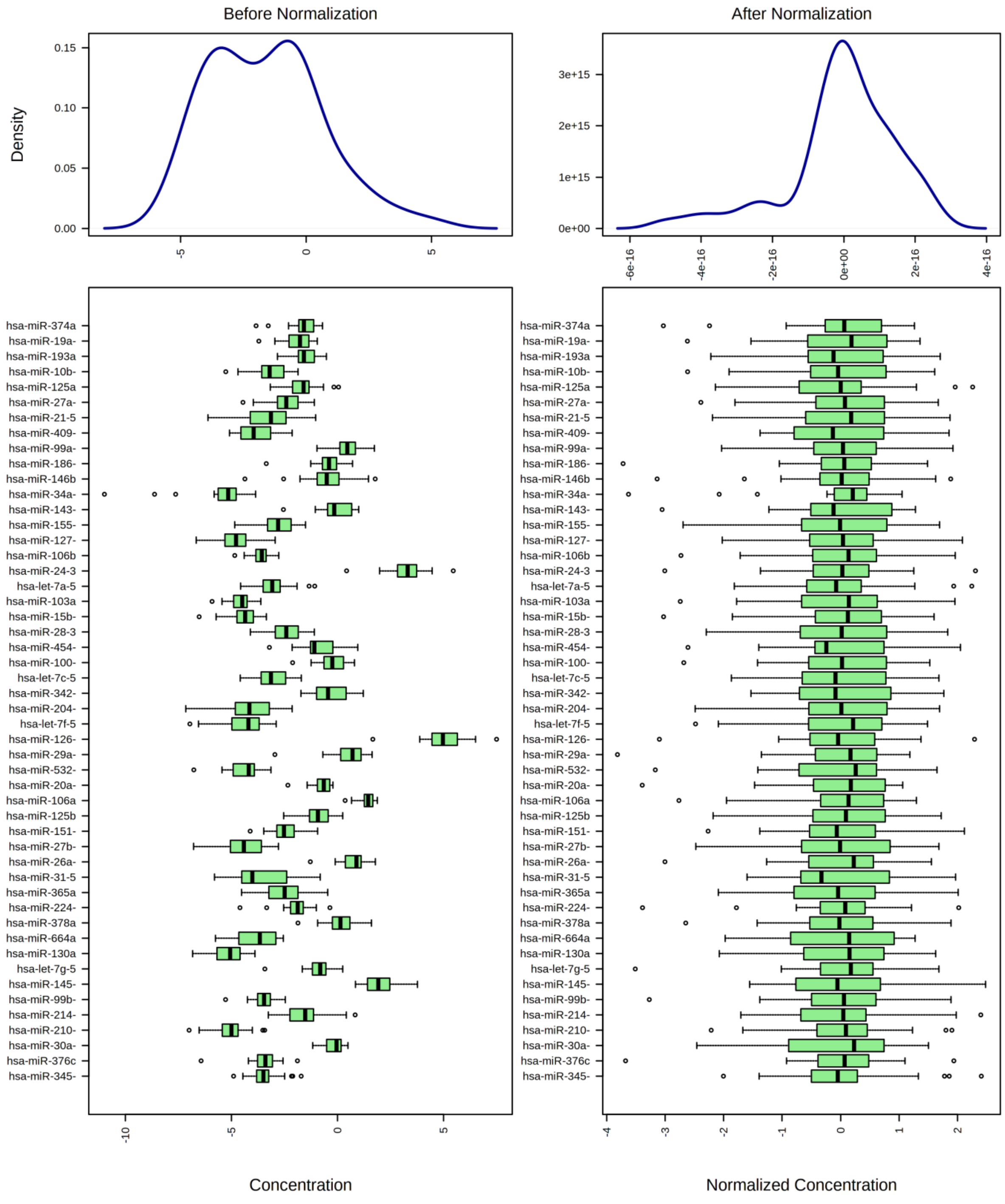

Figure S3. Data autoscaling (feature view) regarding female study subjects (N=26). Density plots involved all features (miRNAs), while boxplots were presented for only top 50 molecules. Data was mean-centered, and divided by the standard deviation (SD) of each variable.

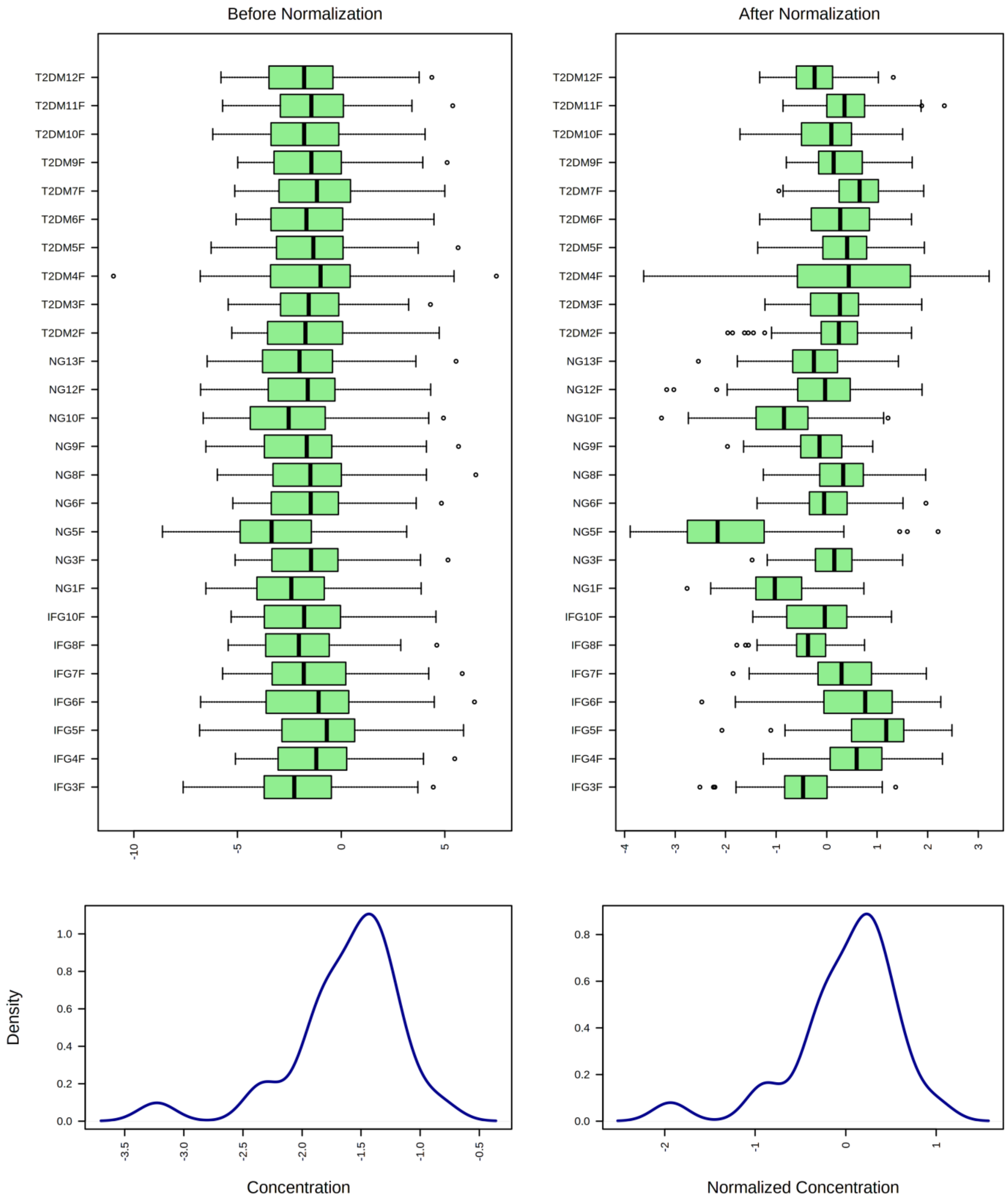

Figure S4. Data autoscaling (sample view) regarding female study subjects (N=26). Density plots and boxplots involved all samples (N=26). Data was mean-centered, and divided by the standard deviation (SD) of each variable. Study subjects were marked dependently on studied groups : diabetic females (T2DMF), prediabetic females (IFGF), normoglycemic females (NGF).

Table S2. Power calculations for all miRNAs for all study subjects (N = 38) and solely for female study participants (N = 26). Results denoted with asterisk (*) were significant after FDR adjustment (0.1).

	**Power observed (alfa=0.05)**	
**miRNAs**	**All subjects (N = 38)**	**Female subjects (N = 26)**
hsa-let-7a-5p	0.73	0.84 *
hsa-let-7b-5p	0.26	0.26
hsa-let-7c-5p	0.39	0.40

hsa-let-7d-5p	0.80	0.97 *
hsa-let-7e-5p	0.71	0.89 *
hsa-let-7f-5p	0.54	0.54
hsa-let-7g-5p	0.14	0.22
hsa-miR-100-5p	0.25	0.78 *
hsa-miR-103a-3p	0.18	0.29
hsa-miR-106a-5p	0.28	0.27
hsa-miR-106b-5p	0.22	0.05
hsa-miR-10a-5p	0.73	0.99 *
hsa-miR-10b-5p	0.57	0.64
hsa-miR-125a-5p	0.24	0.28
hsa-miR-125b-5p	0.83	0.89 *
hsa-miR-126-3p	0.19	0.14
hsa-miR-127-3p	0.85	0.90 *
hsa-miR-130a-3p	0.10	0.15
hsa-miR-132-3p	0.30	0.31
hsa-miR-140-3p	0.21	0.08
hsa-miR-143-3p	0.15	0.07
hsa-miR-145-5p	0.45	0.58
hsa-miR-146a-5p	0.58	0.42
hsa-miR-146b-5p	0.73	0.77 *
hsa-miR-151-3p	0.21	0.14
hsa-miR-152-3p	0.28	0.49
hsa-miR-155-5p	0.79	0.67
hsa-miR-15b-5p	0.32	0.14
hsa-miR-16-5p	0.10	0.06
hsa-miR-186-5p	0.53	0.36
hsa-miR-191-5p	0.59	0.42
hsa-miR-193a-5p	0.06	0.10
hsa-miR-193b-3p	0.21	0.11
hsa-miR-199a-3p	0.68	0.83 *
hsa-miR-19a-3p	0.07	0.09
hsa-miR-204-5p	0.22	0.74 *
hsa-miR-20a-5p	0.15	0.24
hsa-miR-210-3p	0.15	0.18
hsa-miR-21-5p	0.23	0.49
hsa-miR-214-3p	0.14	0.17
hsa-miR-222-3p	0.51	0.50
hsa-miR-224-5p	0.25	0.41
hsa-miR-24-3p	0.21	0.18
hsa-miR-26a-5p	0.50	0.65
hsa-miR-26b-5p	0.27	0.42
hsa-miR-27a-3p	0.05	0.29
hsa-miR-27b-3p	0.08	0.34
hsa-miR-28-3p	0.22	0.28
hsa-miR-29a-3p	0.05	0.08
hsa-miR-30a-3p	0.19	0.16
hsa-miR-30a-5p	0.11	0.13
hsa-miR-30d-5p	0.47	0.40
hsa-miR-30e-3p	0.31	0.23
hsa-miR-31-5p	0.13	0.12

hsa-miR-320a-3p	0.08	0.08
hsa-miR-342-3p	0.75	0.77 *
hsa-miR-345-5p	0.44	0.30
hsa-miR-34a-5p	0.08	0.05
hsa-miR-365a-3p	0.58	0.82 *
hsa-miR-374a-5p	0.21	0.29
hsa-miR-376c-3p	0.36	0.19
hsa-miR-378a-5p	0.09	0.16
hsa-miR-409-3p	0.69	0.73 *
hsa-miR-454-3p	0.55	0.41
hsa-miR-484	0.08	0.12
hsa-miR-532-5p	0.73	0.91 *
hsa-miR-574-3p	0.19	0.38
hsa-miR-664a-3p	0.30	0.32
hsa-miR-708-5p	0.14	0.18
hsa-miR-92a-3p	0.07	0.06
hsa-miR-93-3p	0.24	0.20
hsa-miR-99a-5p	0.73	0.79 *
hsa-miR-99b-5p	0.60	0.56
hsa-miR-140-5p	0.06	0.11
hsa-miR-374-5p	0.41	0.31

Table S3. Spearman correlation coefficients for DE miRNA with FDR adjustment for male and female study participants (N = 38). Results for FI and HOMA-IR were obtained after exclusion of 8 subjects treated with drugs affecting fasting insulin. Bolded records were significant with $p < 0.05$.

miRNA & Anthropometric / Biochemical Parameter	Spearman Correlation Coefficient	*p* Value	FDR
let-7a-5p & BMI	-0,036332	0,828556	0,898695
let-7a-5p & WHR	0,108218	0,517815	0,692034
**let-7a-5p & FPG (mmol/L)**	**0,360274**	**0,02629**	**0,216656**
let-7a-5p & HbA1c [%]	0,273118	0,097098	0,316324
let-7a-5p & TC (mmol/l)	-0,124562	0,456204	0,654843
let-7a-5p & LDL (mmol/L)	-0,214149	0,196689	0,450813
let-7a-5p & HDL (mmol/l)	-0,021022	0,90031	0,942036
let-7a-5p & TG (mmol/L)	0,201806	0,224364	0,481356
let-7a-5p & TG/HDL ratio	0,159208	0,339706	0,571505
let-7a-5p & FI (pmol/L)	0,164405	0,385313	0,605492
let-7a-5p & HOMA-IR	0,224472	0,233053	0,490096
let-7d-5p & BMI	0,444432	0,705489	0,853033
let-7d-5p & WHR	0,340217	0,997399	0,997399
**let-7d-5p & FPG (mmol/L)**	**-0,253174**	**0,005183**	**0,162211**
**let-7d-5p & HbA1c [%]**	**-0,346884**	**0,036619**	**0,216656**
let-7d-5p & TC (mmol/l)	-0,212405	0,125117	0,365138
**let-7d-5p & LDL (mmol/L)**	**0,146211**	**0,032871**	**0,216656**
let-7d-5p & HDL (mmol/l)	0,215013	0,200447	0,450813
let-7d-5p & TG (mmol/L)	0,444432	0,381076	0,605492
let-7d-5p & TG/HDL ratio	0,340217	0,194846	0,450813
let-7d-5p & FI (pmol/L)	0,228031	0,22553	0,481356
let-7d-5p & HOMA-IR	0,313904	0,091168	0,316324
let-7e-5p & BMI	0,340465	0,942818	0,95777

let-7e-5p & WHR	0,251374	0,823999	0,898695
**let-7e-5p & FPG (mmol/L)**	**-0,175241**	**0,036474**	**0,216656**
let-7e-5p & HbA1c [%]	-0,280024	0,127915	0,365837
let-7e-5p & TC (mmol/l)	-0,038211	0,292643	0,53846
let-7e-5p & LDL (mmol/L)	0,124761	0,08861	0,316324
let-7e-5p & HDL (mmol/l)	0,139293	0,819831	0,898695
let-7e-5p & TG (mmol/L)	0,340465	0,455481	0,654843
let-7e-5p & TG/HDL ratio	0,251374	0,40425	0,61713
let-7e-5p & FI (pmol/L)	0,204449	0,278484	0,537112
let-7e-5p & HOMA-IR	0,255617	0,17277	0,426292
miR-10a-5p & BMI	-0,011709	0,944375	0,95777
miR-10a-5p & WHR	0,185907	0,263781	0,523899
**miR-10a-5p & FPG (mmol/L)**	**0,449138**	**0,004675**	**0,162211**
**miR-10a-5p & HbA1c [%]**	**0,417530**	**0,009102**	**0,162211**
**miR-10a-5p & TC (mmol/l)**	**-0,334282**	**0,040243**	**0,216656**
**miR-10a-5p & LDL (mmol/L)**	**-0,428626**	**0,007254**	**0,162211**
miR-10a-5p & HDL (mmol/l)	-0,080911	0,629167	0,789218
miR-10a-5p & TG (mmol/L)	0,138878	0,405666	0,61713
miR-10a-5p & TG/HDL ratio	0,159427	0,339034	0,571505
miR-10a-5p & FI (pmol/L)	0,140823	0,457932	0,654843
miR-10a-5p & HOMA-IR	0,207564	0,271067	0,530995
miR-125b-5p & BMI	-0,175968	0,290612	0,53846
miR-125b-5p & WHR	0,163147	0,327734	0,566781
**miR-125b-5p & FPG (mmol/L)**	**0,333242**	**0,040907**	**0,216656**
miR-125b-5p & HbA1c [%]	0,317924	0,051757	0,245344
**miR-125b-5p & TC (mmol/l)**	**-0,359457**	**0,026658**	**0,216656**
**miR-125b-5p & LDL (mmol/L)**	**-0,432675**	**0,006666**	**0,162211**
miR-125b-5p & HDL (mmol/l)	-0,019160	0,909098	0,942036
miR-125b-5p & TG (mmol/L)	-0,015431	0,926737	0,953406
miR-125b-5p & TG/HDL ratio	-0,007112	0,966196	0,973
miR-125b-5p & FI (pmol/L)	0,164405	0,385313	0,605492
miR-125b-5p & HOMA-IR	0,212458	0,259674	0,523005
miR-127-3p & BMI	-0,121033	0,469163	0,66426
miR-127-3p & WHR	0,247182	0,134612	0,37744
**miR-127-3p & FPG (mmol/L)**	**0,393324**	**0,014566**	**0,162211**
**miR-127-3p & HbA1c [%]**	**0,402594**	**0,012214**	**0,162211**
miR-127-3p & TC (mmol/l)	-0,167798	0,313942	0,561172
miR-127-3p & LDL (mmol/L)	-0,238332	0,149596	0,396152
miR-127-3p & HDL (mmol/l)	0,095911	0,566776	0,730171
miR-127-3p & TG (mmol/L)	0,117756	0,481366	0,668305
miR-127-3p & TG/HDL ratio	0,075610	0,651868	0,803596
miR-127-3p & FI (pmol/L)	0,259622	0,165907	0,423656
miR-127-3p & HOMA-IR	0,305451	0,100708	0,316324
miR-146b-5p & BMI	0,255526	0,12153	0,362058
miR-146b-5p & WHR	0,311741	0,056742	0,245884
**miR-146b-5p & FPG (mmol/L)**	**0,399015**	**0,013081**	**0,162211**
**miR-146b-5p & HbA1c [%]**	**0,424119**	**0,007962**	**0,162211**
miR-146b-5p & TC (mmol/l)	-0,152036	0,362181	0,595309
miR-146b-5p & LDL (mmol/L)	-0,188871	0,256105	0,523005
miR-146b-5p & HDL (mmol/l)	-0,152187	0,361698	0,595309
miR-146b-5p & TG (mmol/L)	0,267141	0,104937	0,319276

miR-146b-5p & TG/HDL ratio	0,298829	0,068384	0,271638
miR-146b-5p & FI (pmol/L)	0,310345	0,095097	0,316324
miR-146b-5p & HOMA-IR	0,346830	0,060422	0,254126
miR-199a-3p & BMI	0,041147	0,806242	0,898695
**miR-199a-3p & WHR**	**0,323340**	**0,047685**	**0,235135**
**miR-199a-3p & FPG (mmol/L)**	**0,407770**	**0,011047**	**0,162211**
**miR-199a-3p & HbA1c [%]**	**0,352298**	**0,030064**	**0,216656**
miR-199a-3p & TC (mmol/l)	-0,244089	0,139717	0,384221
**miR-199a-3p & LDL (mmol/L)**	**-0,398534**	**0,013201**	**0,162211**
miR-199a-3p & HDL (mmol/l)	0,118027	0,480352	0,668305
miR-199a-3p & TG (mmol/L)	0,230150	0,164501	0,423656
miR-199a-3p & TG/HDL ratio	0,092242	0,581771	0,742797
miR-199a-3p & FI (pmol/L)	0,141268	0,456498	0,654843
miR-199a-3p & HOMA-IR	0,203115	0,281702	0,537112
miR-342-3p & BMI	0,062377	0,709867	0,853033
miR-342-3p & WHR	0,194441	0,242088	0,501719
**miR-342-3p & FPG (mmol/L)**	**0,392668**	**0,014746**	**0,162211**
**miR-342-3p & HbA1c [%]**	**0,396445**	**0,013735**	**0,162211**
miR-342-3p & TC (mmol/l)	-0,271344	0,099377	0,316324
**miR-342-3p & LDL (mmol/L)**	**-0,333315**	**0,04086**	**0,216656**
miR-342-3p & HDL (mmol/l)	0,044671	0,790006	0,896594
miR-342-3p & TG (mmol/L)	0,036115	0,829564	0,898695
miR-342-3p & TG/HDL ratio	0,039063	0,81588	0,898695
miR-342-3p & FI (pmol/L)	0,124360	0,512625	0,692034
miR-342-3p & HOMA-IR	0,198220	0,293705	0,53846
hsa-miR-409-3p& BMI	-0,049683	0,767065	0,891822
hsa-miR-409-3p& WHR	0,023088	0,890567	0,942036
hsa-miR-409-3p& FPG (mmol/L)	0,218003	0,188561	0,449404
hsa-miR-409-3p& HbA1c [%]	0,313971	0,054902	0,245344
hsa-miR-409-3p& TC (mmol/l)	-0,169549	0,308845	0,559049
hsa-miR-409-3p& LDL (mmol/L)	-0,208240	0,209623	0,46117
hsa-miR-409-3p& HDL (mmol/l)	0,221164	0,182074	0,441298
hsa-miR-409-3p& TG (mmol/L)	-0,162736	0,328971	0,566781
hsa-miR-409-3p & TG/HDL ratio	-0,164898	0,322499	0,566781
hsa-miR-409-3p & FI (pmol/L)	-0,053615	0,778414	0,891822
hsa-miR-409-3p & HOMA-IR	0,024694	0,89694	0,942036
hsa-miR-532-5p & BMI	-0,046947	0,779565	0,891822
**hsa-miR-532-5p & WHR**	**0,370828**	**0,021907**	**0,208847**
hsa-miR-532-5p & FPG (mmol/L)	0,225773	0,172902	0,426292
hsa-miR-532-5p & HbA1c [%]	0,239734	0,147146	0,396152
hsa-miR-532-5p & TC (mmol/l)	-0,211799	0,201763	0,450813
hsa-miR-532-5p & LDL (mmol/L)	-0,293155	0,074061	0,286234
hsa-miR-532-5p & HDL (mmol/l)	0,108611	0,516285	0,692034
hsa-miR-532-5p & TG (mmol/L)	0,110752	0,507998	0,692034
hsa-miR-532-5p & TG/HDL ratio	0,020024	0,905018	0,942036
hsa-miR-532-5p 8 & FI (pmol/L)	0,064294	0,735712	0,869478
hsa-miR-532-5p & HOMA-IR	0,093660	0,62252	0,787791
hsa-miR-99a-5p & BMI	-0,128365	0,442459	0,654843
hsa-miR-99a-5p & WHR	0,148922	0,37221	0,604841
**hsa-miR-99a-5p & FPG (mmol/L)**	**0,339261**	**0,037184**	**0,216656**
hsa-miR-99a-5p & HbA1c [%]	0,276303	0,09311	0,316324

hsa-miR-99a-5p & TC (mmol/l)	-0,277693	0,091409	0,316324
**hsa-miR-99a-5p & LDL (mmol/L)**	**-0,349182**	**0,031654**	**0,216656**
hsa-miR-99a-5p & HDL (mmol/l)	-0,063612	0,704379	0,853033
hsa-miR-99a-5p & TG (mmol/L)	0,100903	0,546661	0,71718
hsa-miR-99a-5p & TG/HDL ratio	0,075610	0,651868	0,803596
hsa-miR-99a-5p & FI (pmol/L)	0,304561	0,101754	0,316324
hsa-miR-99a-5p & HOMA-IR	0,355729	0,053699	0,245344
hsa-miR-155-5p & BMI	-0,097505	0,560316	0,72841
hsa-miR-155-5p & WHR	0,142357	0,393888	0,61224
hsa-miR-155-5p & FPG (mmol/L)	0,305226	0,062402	0,254956
**hsa-miR-155-5p & HbA1c [%]**	**0,327808**	**0,044522**	**0,227382**
**hsa-miR-155-5p & TC (mmol/l)**	**-0,349278**	**0,031605**	**0,216656**
**hsa-miR-155-5p & LDL (mmol/L)**	**-0,384418**	**0,017176**	**0,175438**
hsa-miR-155-5p & HDL (mmol/l)	-0,101932	0,542555	0,71718
hsa-miR-155-5p & TG (mmol/L)	-0,033488	0,841797	0,90509
hsa-miR-155-5p & TG/HDL ratio	0,048911	0,770583	0,891822
hsa-miR-155-5p & FI (pmol/L)	0,066518	0,726908	0,866232
hsa-miR-155-5p & HOMA-IR	0,145273	0,443702	0,654843

Table S4. Spearman correlation coefficients for DE miRNAs with FDR adjustment for female study participants (N = 26). Results for FI and HOMA-IR were obtained after exclusion of 6 females treated with drugs affecting fasting insulin. Bolded records were significant with $p < 0.05$, while ones underlined passed FDR correction with $p < 0.1$.

miRNA & Anthropometric / Biochemical Parameter	Spearman Correlation Coefficient	*p* Value	FDR
let-7a-5p & BMI	0,083077	0,686598154	0,803819638
let-7a-5p & WHR	0,082393	0,689050683	0,803819638
**let-7a-5p & FPG (mmol/L)**	**0,585841**	**0,001662911**	**0,046552488**
**let-7a-5p & HbA1c [%]**	**0,502065**	**0,008961318**	**0,077821974**
let-7a-5p & TC (mmol/l)	-0,034542	0,866963808	0,917028645
let-7a-5p & LDL (mmol/L)	-0,139169	0,497748261	0,684403859
let-7a-5p & HDL (mmol/l)	0,085602	0,677568468	0,803819638
let-7a-5p & TG (mmol/L)	0,329287	0,10045635	0,290794699
let-7a-5p & TG/HDL ratio	0,231453	0,255256997	0,484108099
let-7a-5p & FI (pmol/L)	0,332331	0,152258789	0,379835034
let-7a-5p & HOMA-IR	0,430075	0,058396006	0,204117973
let-7d-5p & BMI	-0,115897	0,572887074	0,763465583
let-7d-5p & WHR	0,078974	0,701358818	0,803819638
**let-7d-5p & FPG (mmol/L)**	**0,628933**	**0,00057841**	**0,02429223**
**let-7d-5p & HbA1c [%]**	**0,437504**	**0,025407498**	**0,11719259**
let-7d-5p & TC (mmol/l)	-0,217168	0,286573543	0,492548277
let-7d-5p & LDL (mmol/L)	-0,325526	0,10463654	0,297672915
let-7d-5p & HDL (mmol/l)	-0,085602	0,677568468	0,803819638
let-7d-5p & TG (mmol/L)	0,185331	0,364715372	0,557560596
let-7d-5p & TG/HDL ratio	0,200684	0,325601378	0,529161481
let-7d-5p & FI (pmol/L)	0,279699	0,232347861	0,470698082
let-7d-5p & HOMA-IR	0,393985	0,085646481	0,26169758
let-7e-5p & BMI	-0,092650	0,652596421	0,803819638
let-7e-5p & WHR	0,055043	0,789420732	0,862612058
**let-7e-5p & FPG (mmol/L)**	**0,463748**	**0,017020311**	**0,093611711**
let-7e-5p & HbA1c [%]	0,323836	0,106556353	0,29799658
let-7e-5p & TC (mmol/l)	-0,196306	0,33648308	0,539026293

let-7e-5p & LDL (mmol/L)	-0,316635	0,115028886	0,316329437
let-7e-5p & HDL (mmol/l)	0,101695	0,621075122	0,803819638
let-7e-5p & TG (mmol/L)	0,083091	0,686547237	0,803819638
let-7e-5p & TG/HDL ratio	0,076239	0,711258589	0,803819638
let-7e-5p & FI (pmol/L)	0,166917	0,481824918	0,668076568
let-7e-5p & HOMA-IR	0,251128	0,285521711	0,492548277
miR-100-5p & BMI	-0,222564	0,27447103	0,492183466
miR-100-5p & WHR	0,034530	0,867008901	0,917028645
**miR-100-5p & FPG (mmol/L)**	**0,485978**	**0,011832929**	**0,084888404**
miR-100-5p & HbA1c [%]	0,337915	0,091339073	0,27401722
miR-100-5p & TC (mmol/l)	-0,192202	0,346881517	0,539957079
miR-100-5p & LDL (mmol/L)	-0,303984	0,131100612	0,348896791
miR-100-5p & HDL (mmol/l)	-0,052731	0,798082611	0,866339676
miR-100-5p & TG (mmol/L)	0,294409	0,144302941	0,37203102
miR-100-5p & TG/HDL ratio	0,194530	0,340961092	0,539957079
miR-100-5p & FI (pmol/L)	0,330827	0,154236044	0,379835034
miR-100-5p & HOMA-IR	0,425564	0,061384919	0,206704319
miR-10a-5p & BMI	-0,078291	0,70382939	0,803819638
miR-10a-5p & WHR	0,237607	0,24248083	0,470698082
**miR-10a-5p & FPG (mmol/L)**	**0,641929**	**0,000407694**	**0,02429223**
**miR-10a-5p & HbA1c [%]**	**0,545679**	**0,003934021**	**0,052759809**
**miR-10a-5p & TC (mmol/l)**	**-0,437073**	**0,025569292**	**0,11719259**
**miR-10a-5p & LDL (mmol/L)**	**-0,520431**	**0,006418629**	**0,06229846**
miR-10a-5p & HDL (mmol/l)	0,072590	0,724537268	0,813256118
miR-10a-5p & TG (mmol/L)	0,112840	0,58312015	0,769718597
miR-10a-5p & TG/HDL ratio	0,076923	0,708779297	0,803819638
miR-10a-5p & FI (pmol/L)	0,112782	0,635917058	0,803819638
miR-10a-5p & HOMA-IR	0,254135	0,279597636	0,492183466
miR-125b-5p & BMI	-0,026325	0,898425358	0,944204994
miR-125b-5p & WHR	0,264957	0,19083852	0,443497968
miR-125b-5p & FPG (mmol/L)	0,689125	9,88682E-05	0,01631326
**miR-125b-5p & HbA1c [%]**	**0,558728**	**0,003008893**	**0,04964674**
miR-125b-5p & TC (mmol/l)	-0,259918	0,199730461	0,451445563
miR-125b-5p & LDL (mmol/L)	-0,364507	0,067132291	0,213015922
miR-125b-5p & HDL (mmol/l)	0,004451	0,982782179	0,982782179
miR-125b-5p & TG (mmol/L)	0,183963	0,368327909	0,557560596
miR-125b-5p & TG/HDL ratio	0,165128	0,420163033	0,606385356
miR-125b-5p & FI (pmol/L)	0,428571	0,059379774	0,204117973
**miR-125b-5p & HOMA-IR**	**0,529323**	**0,016394084**	**0,093276688**
miR-127-3p & BMI	-0,117265	0,568336323	0,763465583
miR-127-3p & WHR	0,246496	0,224781713	0,469480794
**miR-127-3p & FPG (mmol/L)**	**0,545144**	**0,003976585**	**0,052759809**
**miR-127-3p & HbA1c [%]**	**0,525074**	**0,005882205**	**0,06066024**
miR-127-3p & TC (mmol/l)	-0,064979	0,752480865	0,827728952
miR-127-3p & LDL (mmol/L)	-0,165156	0,420082457	0,606385356
miR-127-3p & HDL (mmol/l)	0,096901	0,637703469	0,803819638
miR-127-3p & TG (mmol/L)	0,296119	0,141878149	0,371585629
miR-127-3p & TG/HDL ratio	0,193162	0,344432345	0,539957079
**miR-127-3p & FI (pmol/L)**	**0,445113**	**0,049225296**	**0,184594861**
**miR-127-3p & HOMA-IR**	**0,538346**	**0,014335462**	**0,090975049**
miR-146b-5p & BMI	0,377094	0,057560271	0,204117973

miR-146b-5p & WHR	0,229402	0,259611217	0,486771031
**miR-146b-5p & FPG (mmol/L)**	**0,572161**	**0,00225709**	**0,046552488**
**miR-146b-5p & HbA1c [%]**	**0,529195**	**0,005438244**	**0,06066024**
miR-146b-5p & TC (mmol/l)	-0,200068	0,327118006	0,529161481
miR-146b-5p & LDL (mmol/L)	-0,262267	0,195550839	0,44813734
miR-146b-5p & HDL (mmol/l)	-0,250300	0,217478111	0,467126372
**miR-146b-5p & TG (mmol/L)**	**0,487605**	**0,011511718**	**0,084888404**
**miR-146b-5p & TG/HDL ratio**	**0,492650**	**0,010561158**	**0,084830243**
**miR-146b-5p & FI (pmol/L)**	**0,578947**	**0,00747966**	**0,068563552**
**miR-146b-5p & HOMA-IR**	**0,636090**	**0,002571107**	**0,047136967**
miR-199a-3p & BMI	0,077607	0,70630289	0,803819638
miR-199a-3p & WHR	0,254017	0,210497434	0,463094355
**miR-199a-3p & FPG (mmol/L)**	**0,577291**	**0,002015887**	**0,046552488**
**miR-199a-3p & HbA1c [%]**	**0,542931**	**0,004156833**	**0,052759809**
miR-199a-3p & TC (mmol/l)	-0,348153	0,081345168	0,253244392
**miR-199a-3p & LDL (mmol/L)**	**-0,491366**	**0,010796576**	**0,084830243**
miR-199a-3p & HDL (mmol/l)	0,043486	0,832942689	0,892438595
miR-199a-3p & TG (mmol/L)	0,289964	0,150746207	0,379835034
miR-199a-3p & TG/HDL ratio	0,167179	0,41433117	0,606385356
miR-199a-3p & FI (pmol/L)	0,187970	0,427421258	0,606385356
miR-199a-3p & HOMA-IR	0,308271	0,186055561	0,438559536
miR-204-5p & BMI	0,248547	0,220823376	0,467126372
miR-204-5p & WHR	0,383248	0,053284991	0,195378301
**miR-204-5p & FPG (mmol/L)**	**0,628249**	**0,000588903**	**0,02429223**
**miR-204-5p & HbA1c [%]**	**0,458452**	**0,018498046**	**0,098457341**
**miR-204-5p & TC (mmol/l)**	**-0,390561**	**0,048531575**	**0,184594861**
**miR-204-5p & LDL (mmol/L)**	**-0,475979**	**0,013974978**	**0,090975049**
miR-204-5p & HDL (mmol/l)	-0,278720	0,167954925	0,401631342
**miR-204-5p & TG (mmol/L)**	**0,434604**	**0,026510174**	**0,118221047**
**miR-204-5p & TG/HDL ratio**	**0,401026**	**0,042316231**	**0,166242336**
**miR-204-5p & FI (pmol/L)**	**0,418045**	**0,066621562**	**0,213015922**
**miR-204-5p & HOMA-IR**	**0,529323**	**0,016394084**	**0,093276688**
miR-342-3p & BMI	0,021538	0,916823731	0,957442503
miR-342-3p & WHR	0,243761	0,230132875	0,470698082
**miR-342-3p & FPG (mmol/L)**	**0,446648**	**0,02217144**	**0,110857199**
**miR-342-3p & HbA1c [%]**	**0,432353**	**0,027392422**	**0,118451131**
**miR-342-3p & TC (mmol/l)**	**-0,409029**	**0,038002318**	**0,156759562**
**miR-342-3p & LDL (mmol/L)**	**-0,430843**	**0,02799754**	**0,118451131**
miR-342-3p & HDL (mmol/l)	0,100325	0,625808084	0,803819638
miR-342-3p & TG (mmol/L)	-0,010600	0,959012233	0,973460707
miR-342-3p & TG/HDL ratio	-0,009915	0,961661183	0,973460707
miR-342-3p & FI (pmol/L)	-0,009023	0,969885	0,975798933
miR-342-3p & HOMA-IR	0,133835	0,573755953	0,763465583
miR-365a-3p & BMI	-0,184957	0,365699971	0,557560596
miR-365a-3p & WHR	0,238974	0,239699912	0,470698082
**miR-365a-3p & FPG (mmol/L)**	**0,475718**	**0,014034813**	**0,090975049**
**miR-365a-3p & HbA1c [%]**	**0,454331**	**0,019719169**	**0,101676966**
miR-365a-3p & TC (mmol/l)	-0,219904	0,280395429	0,492183466
miR-365a-3p & LDL (mmol/L)	-0,239357	0,238925257	0,470698082
miR-365a-3p & HDL (mmol/l)	0,212635	0,29699779	0,49892482
miR-365a-3p & TG (mmol/L)	0,082749	0,687773295	0,803819638

miR-365a-3p & TG/HDL ratio	0,012650	0,951096022	0,973460707
miR-365a-3p & FI (pmol/L)	0,102256	0,667935357	0,803819638
miR-365a-3p & HOMA-IR	0,204511	0,387090238	0,580635357
hsa-miR-409-3p& BMI	-0,016068	0,93790108	0,969178237
hsa-miR-409-3p& WHR	0,070085	0,733698246	0,81377545
hsa-miR-409-3p& FPG (mmol/L)	0,152189	0,457973324	0,640386428
hsa-miR-409-3p& HbA1c [%]	0,204329	0,316705185	0,522563556
hsa-miR-409-3p& TC (mmol/l)	-0,225718	0,267550237	0,490508768
hsa-miR-409-3p& LDL (mmol/L)	-0,235254	0,247315164	0,474500024
hsa-miR-409-3p& HDL (mmol/l)	0,214690	0,292244502	0,497116937
hsa-miR-409-3p& TG (mmol/L)	-0,221234	0,27742265	0,492183466
hsa-miR-409-3p & TG/HDL ratio	-0,211624	0,299354892	0,49892482
hsa-miR-409-3p & FI (pmol/L)	-0,094737	0,691152596	0,803819638
hsa-miR-409-3p & HOMA-IR	0,018045	0,9398092	0,969178237
hsa-miR-532-5p & BMI	0,109060	0,595879704	0,78031866
hsa-miR-532-5p & WHR	0,331966	0,097556127	0,287442158
**hsa-miR-532-5p & FPG (mmol/L)**	**0,527360**	**0,005632312**	**0,06066024**
**hsa-miR-532-5p & HbA1c [%]**	**0,443686**	**0,023180955**	**0,112495811**
hsa-miR-532-5p & TC (mmol/l)	-0,069767	0,734863891	0,81377545
hsa-miR-532-5p & LDL (mmol/L)	-0,167208	0,41425022	0,606385356
hsa-miR-532-5p & HDL (mmol/l)	0,049307	0,810953573	0,874557775
hsa-miR-532-5p & TG (mmol/L)	0,365533	0,06630913	0,213015922
hsa-miR-532-5p & TG/HDL ratio	0,241709	0,234201422	0,470698082
hsa-miR-532-5p 8 & FI (pmol/L)	0,157895	0,506146584	0,690199887
hsa-miR-532-5p & HOMA-IR	0,261654	0,26512411	0,490508768
hsa-miR-99a-5p & BMI	-0,094701	0,645394612	0,803819638
hsa-miR-99a-5p & WHR	0,249915	0,218210625	0,467126372
**hsa-miR-99a-5p & FPG (mmol/L)**	**0,577633**	**0,002000624**	**0,046552488**
**hsa-miR-99a-5p & HbA1c [%]**	**0,469785**	**0,015455503**	**0,093276688**
hsa-miR-99a-5p & TC (mmol/l)	-0,164159	0,422935001	0,606385356
hsa-miR-99a-5p & LDL (mmol/L)	-0,255770	0,207258411	0,462130242
hsa-miR-99a-5p & HDL (mmol/l)	0,087656	0,670253209	0,803819638
hsa-miR-99a-5p & TG (mmol/L)	0,285177	0,157911756	0,383168231
hsa-miR-99a-5p & TG/HDL ratio	0,161709	0,429982344	0,606385356
hsa-miR-99a-5p & FI (pmol/L)	0,351880	0,128141952	0,346613477
**hsa-miR-99a-5p & HOMA-IR**	**0,464662**	**0,039002619**	**0,156961758**

Table S5. ROC curves values characteristics generated among IFG and NG female subjects and T2DM and NG females.

	**IFG vs NG Female Subjects**	
**miRNA**	**AUC**	***p* Value**
miR-199a-3p	0.911	0.009
miR-204-5p	0.889	0.006
miR-127-3p	0.878	0.003
miR-146b-5p	0.878	0.01
miR-342-3p	0.878	0.007
miR-125b-5p	0.867	0.008
miR-532-5p	0.867	0.01
let-7e-5p	0.856	0.004
miR-99a-5p	0.811	0.01
miR-365a-3p	0.8	0.012

miR-100-5p	0.778	0.022
	**T2DM vs NG Female Subjects**	
**miRNA**	**AUC**	***p* Value**
miR-365a-3p	0.889	0.005
miR-532-5p	0.905	0.007
miR-409-3p	0.889	0.004
let-7e-5p	0.857	0.01
miR-199a-3p	0.873	0.0496
miR-99a-5p	0.873	0.023
miR-127-3p	0.841	0.006
let-7a-5p	0.841	0.019
miR-100-5p	0.841	0.021
miR-342-3p	0.841	0.025
